# Cattle Immunized with a Recombinant Subunit Vaccine Formulation Exhibits a Trend towards Protection against *Histophilus somni* Bacterial Challenge

**DOI:** 10.1371/journal.pone.0159070

**Published:** 2016-08-08

**Authors:** Claudia Avis Madampage, Don Wilson, Hugh Townsend, Gordon Crockford, Neil Rawlyk, Donna Dent, Brock Evans, Joyce Van Donkersgoed, Craig Dorin, Andrew Potter

**Affiliations:** 1 Vaccine and Infectious Disease Organization-International Vaccine Centre, University of Saskatchewan, 120 Veterinary Road, Saskatoon, Saskatchewan, S7N 5E3, Canada; 2 Alberta Beef Health Solutions Inc, Box 307, Picture Butte, Alberta, T0K 1V0, Canada; 3 Veterinary Agri-Health Services, 201–151 East Lake Blvd, Airdrie, Alberta, T4A 2G1, Canada; National Institute of Animal Biotechnology, INDIA

## Abstract

Histophilosis, a mucosal and septicemic infection of cattle is caused by the Gram negative pathogen *Histophilus somni* (*H*. *somni*). As existing vaccines against *H*. *somni* infection have shown to be of limited efficacy, we used a reverse vaccinology approach to identify new vaccine candidates. Three groups (B, C, D) of cattle were immunized with subunit vaccines and a control group (group A) was vaccinated with adjuvant alone. All four groups were challenged with *H*. *somni*. The results demonstrate that there was no significant difference in clinical signs, joint lesions, weight change or rectal temperature between any of the vaccinated groups (B,C,D) *vs* the control group A. However, the trend to protection was greatest for group C vaccinates. The group C vaccine was a pool of six recombinant proteins. Serum antibody responses determined using ELISA showed significantly higher titers for group C, with *P* values ranging from < 0.0148 to < 0.0002, than group A. Even though serum antibody titers in group B (5 out of 6 antigens) and group D were significantly higher compared to group A, they exerted less of a trend towards protection. In conclusion, the vaccine used in group C exhibits a trend towards protective immunity in cattle and would be a good candidate for further analysis to determine which proteins were responsible for the trend towards protection.

## 1. Introduction

*Histophilus somni*, previously known as *Haemophilus somnus*, is an economically important pathogen that affects the cattle industry by causing a variety of mucosal and systemic infections [1–5], including septicemia, respiratory disease, reproductive tract disorders, pericarditis, pleuritis, infectious thrombotic meningoencephalitis (ITME), myocarditis, and arthritis [5–9]. *H*. *somni* is also associated with the bovine respiratory disease (BRD) complex, a leading cause of illness and death in the cattle industry [10]. The most common factors contributing to virulence include interaction of *H*. *somni* with bovine mononuclear phagocytic cells that results in inhibition of phagocytic cell function, attachment to non-epithelial and bovine aortic endothelial host cells, uptake of iron and other nutrients from the host, and antigenic variation of *H*. *somni* outer membrane proteins (OMPs) [6,11,12]. Additionally, expression of high-molecular-weight surface immunoglobulin binding protein A (IbpA), that binds the Fc portion of IgG2, increases *H*. *somni* serum resistance and, sialyation (addition of neuraminic acid) of lipooligosaccharides (LOS) may hinder antibody binding to certain epitopes increasing serum resistance [13–16]. Also, under favorable growth conditions the formation of branched mannose-galactose exopolysaccharide polymers for biofilm formation may contribute towards the virulence of *H*. *somni* [13–16]. Previously, Corbeil and co-workers assessed antibody responses to three immunoglobulin binding proteins (IgBPs) such as (IbpA3, IbpA5, IbpA DR2) encoded by the 12.2 Kb gene *ibpA*. The results implied that IbpA DR2 may be a protective antigen [17]. Additionally, the same group used subcutaneous immunization followed by *H*. *somni* challenged (intrabronchially) that showed for the first time protection in a natural host [18].

Vaccination has proven to be the most cost-effective intervention in protecting animals from infectious diseases and increasing livestock productivity [19]. The present day vaccines for *H*. *somni* associated disease have limited efficacy based, in part, to the strategies used by this pathogen to evade host immunity [5,20,21]. Commercial vaccines for *H*. *somni* include killed cells or outer membrane proteins that have helped prevent ITME and pneumonia [22,23]. Reverse vaccinology coupled with modern bioinformatics and next generation whole genome sequencing techniques has opened a pathway to identify reservoirs of genes that encode all surface exposed proteins that are more likely to be potential antigenic vaccine candidates [24]. The development of vaccines using a reverse vaccinology strategy introduces a robust *in silico* method of analyzing the entire genome of the pathogen to identify genes that encode proteins that are surface-localized and could potentially elicit an immune response [25–28]. These proteins can be further classified based on their antigenicity (surface exposed, signal peptides, and B-cell epitopes) [27–29]. Surface exposed proteins (e.g. outer membrane proteins) are considered good vaccine candidates since they have the capacity to induce an immune response following natural infection [27,28]. Reverse vaccinology has the added advantage of identifying a large number of target gene products that may induce the desired immunogenicity in a shorter length of time compared to traditional vaccinology approaches [27,28]. This was proven for serogroup B *Neisseria meningitidis* (MenB) where nearly 600 potential vaccine candidates were identified in a short period of 18 months compared to 40 years of conventional vaccinology [27,28]. Additionally, reverse vaccinology also holds the prospect of identifying novel proteins that may set the stage for the discovery of new host-pathogen interactions, new multivalent vaccine antigens, and the development of novel vaccines with long-term protective immunity [25–30].

The limited efficacy of most current *H*. *somni* vaccines could be due to many reasons and have been explained in references [16,31,32,33]. For example, present vaccines may only address the planktonic form and overlook other bacterial profiles (e.g. *H*. *somni* may form biofilms during myocarditis) [16,31,33]. Also, vaccines manufactured under artificial conditions may not represent the true antigenic profile of *H*. *somni* in the host [33]. Recently, small non-coding RNAs (sRNAs) identified in pathogenic *H*. *somni* strain 2336 (NCBI, GenBank accession number NC_010519) may suggest strain specific variation that in turn may affect protection through vaccination [32]. Finally, in this present study we hypothesize that current *H*. *somni* bacterial strains circulating in the field may be different from strains used for existing vaccines (based on bacterial isolates from the 1980s) [33,34]. Based on the information from previous studies [33,34], we applied a reverse vaccinology strategy and tested vaccine induced immunity using a large animal model (bovine).

## 2. Materials and Methods

### 2.1 Bacterial strains and Growth conditions

As previously stated [33,34], the new isolates of *H*. *somni* were collected from Alberta feedlot calves that died during 2012–13 while strains from the 1980s were stored in -80°C at VIDO-InterVac. The tissue samples from Alberta feedlot calves were obtained from swabs (Amies transport with charcoal, used for collecting, transporting and maintenance of microorganisms) of heart, lung, liver and synovial fluid that were cultured for *H*. *somni* [33,34]. TSA-Blood Agar plates were streaked and incubated for 24–48 hours in 5% CO_2_ at 37°C. Overnight growth of bacteria was accomplished at 37°C (with shaking) by inoculating a single colony of *H*. *somni* in brain heart infusion broth containing 0.1% trizma base, and 0.01% thiamine monophosphate (BHITT). Stocks of the overnight culture were made using 30% heat inactivated fetal calf serum in 15% glycerol and stored at -80°C [33,34]. As previously described [33], PCR of the 16S ribosomal RNA gene was carried out for verification of *H*. *somni*. Plates containing *H*. *somni* bacterial growth were scraped and transferred to BHITT for overnight growth at 37°C (with shaking). Genomic DNA isolation was carried out with cell pellets collected from overnight cultures using a Qiagen genomic DNA extraction kit (Qiagen genomic-tip as described by the manufacturer; Qiagen Canada, 181 Bay Street, Suite 4400, Toronto, Ontario M5J 2T3) followed by electrophoresis on a 1% agarose gel [33,34].

### 2.2 Genome sequencing, *de novo* assembly of next generation sequencing (NGS), antigen prediction, and ranking of outer membrane proteins (OMPs)

As described previously [33,34], genomic DNA from 12 *H*. *somni* isolates that included six new isolates (year, 2012–2013) and six old isolates (year, 1980s) were sequenced using Illumina Miseq with paired end 150 bp read type, at Cofactor genomics (Cofactor genomics, 4044 Clayton Avenue, Saint Louis, Missouri, 63110, USA). The methods used for *de novo* assembly of Illumina reads, antigen prediction, and ranking of OMPs have been described in reference [34–39]. A single new strain (AVI1) was selected as the template for cloning in *Escherichia coli* (*E*. *coli*) based on the rank of its proteins which were also conserved between all 12 isolates [34].

### 2.3 Cloning, expression, and protein purification

In order to test the protective efficacy against *H*. *somni* related septicemia, myocarditis, and arthritis AVI1 antigens were used as multiple subunit vaccines in a bovine vaccination and *H*. *somni* challenge model. Two animal trials comprising 40 (Trial # 1) or 32 (Trial # 2) cattle, respectively, were carried out. Here we publish the results from trial # 2 which included 4 groups of 8 animals each.

As was described earlier [34], a new *H*. *somni* strain (AVI1) was selected and used for amplification of genes using PCR (rank of each gene or protein is denoted by “R”) for animal trial # 2 (**Table 1**). Antigens, R1, R2, R5, R8, R13, R15 and R18 were previously described in reference [34] and were also used in trial # 1. PCR amplification (initial denaturation at 98°C for 30 seconds, denaturation at 98°C for 10 seconds, annealing at 62–65°C for 30 seconds, extension at 72°C for 2 minutes, and a final extension at 72°C for 5 minutes) of the rest of the antigens used in trial # 2 was also performed in a PTC-100 Thermal Cycler (Bio-Rad Laboratories, Hercules, California, USA) with 30 cycles [33,34]. The DNA sequences and amino acid sequences of antigens used in animal trial # 2 have been stated in (**S1 Table).** PCR products of R1, R2, R8, R18, R15, R34, and R35 were double digested with restriction enzyme pairs, (BglII, NcoI), or (XmaI, NcoI) as stated in **Table 1**, and inserted into an N-terminal hexa-histidine affinity tag in-house cloning vector pGH433His.2 [34,40] downstream of an Isopropyl-β-d-thiogalactopyranoside (IPTG) inducible tac promoter. As previously stated [34], pGH433 which is identical to pGH433His.2 except for the exclusion of the histidine tag, was used on double digested PCR products R5 and R13 using restriction enzyme pair (BglII, NcoI) [34,40]. Another in-house cloning vector pAA352 [41], was used on PCR products R4, R21, R23, R24, R27, R35, R36, and R37. The genes inserted into pAA352 via (BamHI, NcoI) restriction sites expressed a leukotoxin (Lkt) fusion protein [41]. The Lkt-antigen(R) fusion protein had the antigen expressed as a C-terminal fusion relative to Lkt [41]. Lkt alone is expressed as a 99259 Da molecular weight protein [41]. Vectors, pGH433, pGH433His.2 and pAA352 all contain an ampicillin resistance gene. All DNA sequences were verified by dideoxy DNA sequencing.

**Table 1:** Cloning details for vaccine groups B, C and D: Primer sequences, PCR product size, vectors used, molecular weights of antigens, and blastp comparison on NCBI. “R” is denoted for rank of gene or protein. The vaccine for the control group A did not contain antigens.

Vaccine	primer pair name	primer sequence 5'→3'	PCR product size (bp)	PCR product-enzyme DD digestion	Vector	Vector- enzyme DD digestion	Molecular weight of ranked protein (Da)	protein purification technique	blastp comparison number on NCBI, (%) & protein details
**Group B**	** **	** **	** **	** **	** **	** **	** **	** **	
**R2**	AVI1R2-F	CACCCCGGGGATACTGAATCACCGAGTAGCAAT	2847	XmaI, NcoI	pGH433His.2 (His-tag)	XmaI,NcoI	109487.84	Ni-NTA agarose	ACA32419.1, (97%): TonB-dependent lactoferrin and transferrin receptor in *H*. *somni* 2336
	AVI1R2-R	CACCCATGGTTAAAACTTCATTTCCATACTCACGGTAAAGT							
**R5**	AVI1R5-F	CACCAGATCTATGAAAAAAACAATTATTGCATTATCTATCG	1048	BglII, NcoI	pGH433 (no -tag)	BglII,NcoI	36697.82	aggregate preparation	ABI25310.1, (96%):Hemaggilutinin antigen in Haemophilus somnus 129PT
	AVI1R5-R	CACCCATGGTTATTTGCTACCTTTCACTGAGATT							
**R8**	AVI1R8-F	CACCAGATCTATGAAAAAACTGTTAATTGCAAGCCT	2434	BglII, NcoI	pGH433His.2 (His-tag)	BglII,NcoI	89312.9	Ni-NTA agarose	WP01234060.1, (97%):surface antigen (D15) [Histophilus somni]
	AVI1R8-R	CACCCATGGCTAAAAAGAACCACCAATACTAAATTG							
**R18**	AVI1R18-F	CACCAGATCTTGTGGTAATTTAAGTAATGTCACCG	787	BglII, NcoI	pGH433His.2 (His-tag)	BglII,NcoI	31059.81	Ni-NTA agarose	WP011608995.1, (99%):plastocyanin [Histophilus somni] OR AAA2941.1, (99%): 31KDa antigen [Histophilus somni]
	AVI1R18-R	CACCCATGGTTATTTTTCGTAGTATAATGATGGACCAGTTG							
**R27**	fus-F-Lkt-R27	CGCGGATCCGATATATACAGTGGTAATGTAT	414	BamHI, NcoI	pAA352 (Lkt fusion)	BamHI, NcoI	15284.64	Lkt-fusion protein (aggregate preparation)	WP011609106.1, (100%): membrane protein [Histophilus somni]
	fus-R-Lkt-R27	CACCCATGGCTACATTACAGAAACATTTAAAGTGG							
**R37**	fus-F-LKT-R37	CGCGGATCCCAAGCTCAACAATCAAATAGTAGTAATA	1134	BamHI, NcoI	pAA352 (Lkt fusion)	BamHI, NcoI	39995.26	Lkt-fusion protein (aggregate preparation)	WP012340387.1, (88%): Hemaggilutinin [Histophilus somni]
	fus-R-LKT-R37	CACCCATGGGAATTAGTTAAACACACCGCTGACG							
**Group C**									
**R13**	AVI1R13-F	CACCAGATCTACAACTGTTTATAATCAAAACGGTACC	1114	BglII, NcoI	pGH433 (no -tag)	BglII, NcoI	41933.1	aggregate preparation	WP012340590.1, (89%): *H*. *somni* porin
	AVI1R13-R	CACCCATGGTTAGAAGTAAACACGTAAGCCTGC							
**R15**	AVI1R15-F	CACCCCGGGAACTTACAACAGCAATGTTTGA	2292	XmaI, NcoI	pGH433His.2 (His-tag)	XmaI, NcoI	91175	Ni-NTA agarose	WP012341555.1, (99%): LPS-assembly protein LptD in *H*. *somni*
	AVI1R15-R	CACCCATGGTTAGTATAGACTAAAGGCTTTAAT							
**R21**	fus-F-Lkt-R21	CGCGGATCCCATGAAGCAGGCAGTTTTATTG	609	BamHI, NcoI	pAA352 (Lkt fusion)	BamHI, NcoI	23530.26	Lkt-fusion protein (aggregate preparation)	WP011609419.1, (93%): membrane protein in *H*. *somni*
	AVIR21-R	CACCCATGGTTAGAATTTCCAACCTAAACC							
**R24**	fus-F-Lkt-R24	CGCGGATCCGCAGCTTTCCAACTCGCCGAG	192	BamHI, NcoI	pAA352 (Lkt fusion)	BamHI, NcoI	9078.53	Lkt-fusion protein (aggregate preparation)	ACA32123.1, (99%): outer membrane protein P1 [Haemophilus somnus 2336]
	AVI1R24-R	CACCCATGGTTATATTAATTCTAGATTGAACGTATACCC							
**R34**	AVI1R34-F	CACCAGATCTGAAACAAGTAAAAATAAAGTTGAAC	1420	BglII, NcoI	pGH433His.2 (His-tag)	BglII, NcoI	54865.75	Ni-NTA agarose	ABI25169.1, (99%): conserved hypothetical protein [Haemophilus somnus 129PT]
	AVI1R34-R	CACCCATGGTCACGCTCTAAACGTAACCCC							
**R36**	fus-F-Lkt-R36	CGCGGATCCCACAGTCCCAAAAGTCCGAGCGAACA	1423	BamHI, NcoI	pAA352 (Lkt fusion)	BamHI, NcoI	55766.68	Lkt-fusion protein (aggregate preparation)	WP011608601.1, (99%): hypothetical protein in *H*. *somni*
	AVI1R36-R	CACCCATGGTCAAAACTGCTTATTAATTTCCAGAAAC							
**Group D**									
R1	AVI1R1-F	CACCAGATCTATGCTTTCATCTACGTTTTATACAAG	2158	BglII, NcoI	pGH433His.2 (His-tag)	BglII, NcoI	81916.3	Ni-NTA agarose	WP036840687.1, (53%): TonB-dependent receptor [Photorhabdus temperata]
	AVI1R1-R	CACCCATGGTTAAAATTTCATTGAAATACTCATCTTAACATT							
R4	fus-F-Lkt-R4	CGCGGATCCATGTTAAAACTTTCTAAAATGACGTTTGCA	1404	BamHI, NcoI	pAA352 (Lkt fusion)	BamHI, NcoI	51838.38	Lkt-fusion protein (aggregate preparation)	WP011608899.1, (98%): membrane protein in *H*. *somni*
	AVI1R4-R	CACCCATGGTTAGCGAGGGGAATTAGTAGAATA							
**R22**	fus-F-Lkt-R22	CGCGGATCCTCTGCTTTCCAACTCGCCGAG	177	BamHI, NcoI	pAA352 (Lkt fusion)	BamHI, NcoI	7898	Lkt-fusion protein (aggregate preparation)	ACA32169.1, (100%): outer membrane protein P1 [Haemophilus somnus 2336]
	AVI1R22-R	CACCCATGGTCAACGTATACCCCCCCCCCATGAG							
**R23**	fus-F-Lkt-R23	CGCGGATCCAGTGATAATATCGCTGTTGTT	541	BamHI, NcoI	pAA352 (Lkt fusion)	BamHI, NcoI	21582.86	Lkt-fusion protein (aggregate preparation)	WP012340600.1, (99%): membrane protein in *H*. *somni*
	AVI1R23-R	CACCCATGGTTACTTAGCCTTTGCTGGAGC							
**R35**	AVI1R35-F	CACCAGATCTGGGGGGGGGGGGAATTTCAAC	709	BglII, NcoI	pGH433His.2 (His-tag)	BglII, NcoI	27974.81	Ni-NTA agarose	ACA31830.1, (87%):conserved hypothetical protein [Haemophilus somnus 2336]
	AVI1R35-R	CACCCATGGTTAGAATTTAAAGCCAAGCGTTAAG							

Transformation of plasmids into *E*. *coli* DH5αF’Iq was performed using conventional techniques [34]. For protein expression, an overnight sterile 20 mL Luria-Bertani (LB) medium + ampicillin (100 μg/mL) with *E*. *coli* DH5αF’Iq containing plasmid pGH433-antigen(R) or pGH433His.2-antigen(R) or pAA352-antigen(R) was transferred to 1 liter of LB medium containing 100 μg/mL ampicillin and grown at 37°C (with shaking) until the optical density (OD) at 600 nm reached 0.6. Protein expression was induced with IPTG (catalog No: I5502, Sigma) at a final concentration of 1 mM with further incubation for 3–4 hours at 37°C (with shaking). The cells were harvested by centrifugation at 10,000 × g for 15 min at 4°C and the pellet resuspended in lysis buffer (50 mM NaH_2_PO_4_, 300 mM NaCl, 10 mM imidazole at pH 8.0) for proteins having histidine tag. For proteins which need to be purified as aggregate or Lkt fusions, the cell pellet was resuspended in 50 mM Tris containing 25% sucrose at pH 8.0 and frozen at -80°C for 20 minutes (**Table 1**).

Antigens fused with an N-terminal histidine tag (R1, R2, R8, R15, R18, R34, R35) were purified as described in reference [34]. As mentioned earlier, cell pellets collected for inclusion body preparations (antigens: R5, R13) or LktA fusion proteins (R4, R21, R22, R23, R24, R27, R36,R37) were resuspended in 50 mM Tris containing 25% sucrose at pH 8.0 and frozen at -80°C for 20 minutes. These cells were lysed using lysozyme (1 mg/mL) and stored on ice for 15 minutes. Next, RIPA (20 mM Tris at pH 7.5, 300 mM NaCl, 2% w/v deoxycholic acid, 2% w/v Nonidet P-40)) and TET (100 mM Tris at pH 8.0, 50 mM EDTA at pH 8.0, 2% w/v Triton X-100) were added at a 5:4 ratio respectively and the cell lysate gently vortexed for 20 seconds. The lysate was further stored on ice for 5 minutes. The bacteria were sonicated and the cell pellet collected by centrifugation at 10,000 × g for 30 min at 4°C. The inclusion bodies were solubilized in 8 M urea containing 100 mM NaH_2_PO_4_ and 10 mM Tris at pH 8.0. Proteins (5–10 μL) were analyzed using 10–12% SDS-PAGE gels in an equal volume of 2 × Laemmli SDS-PAGE loading dye containing 0.5% v/v β-mercaptoethanol. Molecular weights of the predicted proteins are stated in **Table 1** [34].

### 2.4 Experimental animals

Healthy 8–10 month old cattle were obtained from a commercial ranch in Saskatchewan, Canada. All animals were screened for the presence of *H*. *somni*-specific antibodies prior to the trial. Cattle were housed outdoors under feedlot conditions and fed limited barley based rations and free-choice hay. Cattle were randomly assigned to four groups of 8 animals each. All experiments were approved by the University Committee on Animal Care and Supply (University Animal Care Committee, Animal Research Ethics Board, University of Saskatchewan, protocol # 20150001).

### 2.5 Vaccination and challenge of cattle

#### 2.5.1 Vaccine groups

Cattle were randomly assigned to four groups (groups, A, B, C, & D) of 8 animals each. Group A was the control group of animals that received a formulation containing adjuvant alone. Cattle in groups B, C, and D were immunized with antigen pools consisting of multiple proteins (see below). All four groups were challenged with *H*. *somni*.

#### 2.5.2 Vaccine formulation

All vaccines were formulated at VIDO-InterVac, Saskatoon and delivered in a 2 mL dose via the subcutaneous route with two immunizations being given four weeks apart. Vaccines were coded so that the experimental team was blinded to the composition of each formulation and group. Each vaccine dose contained 100 μg of the appropriate antigen [Group B: R2 (100 μg), R5 (100 μg), R8 (100 μg), R18 (100 μg), R27 (100 μg), R37 (100 μg) = total 600 μg’s of antigens; Group C: R13 (100 μg), R15 (75 μg), R21 (100 μg), R24 (100 μg), R34 (100 μg), R36 (100 μg) = total 575 μg’s of antigens; Group D: R1 (100 μg), R4 (100 μg), R22 (100 μg), R23 (100 μg), R35 (100 μg) = total 500 μg’s of antigens], except for antigen R15 which contained 75 μg due to low protein yield. Group A, the control group, only received the Emulsigen Plus (MVP Laboratories, Omaha, NE, USA) supplemented with CpG2007 (lot # NBZ5347/08) [42]. The composition of each vaccine used in the animal trial is described in **Table 2**.

**Table 2:** Vaccine formulation for a single 2 mL dose. “R” is denoted for rank of gene or protein. The vaccine for group A (placebo/control group) did not contain antigens. The vaccine for groups B, C and D contained antigens (R2, R5, R8, R18, R27, R37), (R13, R15, R21, R24, R34, R36) and (R1, R4, R22, R23, R35) respectively. Emulsigen and CpG oligodeoxynucleotides were used together to achieve a balanced Th1/Th2 immune response.

	Group A (control)	Group B vaccine	Group C vaccine	Group D vaccine
Emulsigen (μl)	600	600	600	600
CpG—adjuvant (μl)	250	250	250	250
0.1 M PBS (μl)	1150	Adjust accordingly	Adjust accordingly	Adjust accordingly
Antigen (100 μg)		R2	R13	R1
		R5	R15*	R4
		R8	R21	R22
		R18	R24	R23
		R27	R34	R25
		R37	R36	

* The antigen R15 had a total concentration of 75 μg only.

#### 2.5.3 *H*. *somni* preparation for bacterial challenge

Initial immunization of animals was followed by a booster injection after 28 days. Each animal was challenged with 7.5 x 10^8^ CFU *H*. *somni* (AVI1) 42 days after initial immunization via intravenous inoculation. The animals were euthanized on day 63. After the *H*. *somni* challenge all animals were monitored on a daily basis for changes in body weight, body temperature and clinical signs of disease (depression, lameness, and respiratory distress).

#### 2.5.4 Clinical examination

All animals were monitored on a daily basis for changes in body weight, body temperature and clinical signs of disease (depression, lameness, and respiratory distress) for three weeks post *H*. *somni* challenge. A clinical scoring points system (from 0 to 4) was used for assessing depression, lameness and respiratory distress. Clinical scores for the assessment of depression were based on the following scale: 0 = bright alert (ears erect, eyes bright, chews cud, stays with group, eating, drinking); 1 = mildly depressed (ears may droop, attempts to stay with group, difficult to corner, eating, drinking); 2 = depressed (walks slowly, lethargic, stands alone with head low, easy to corner, appetite decrease, come to eat but not aggressively); 3 = severely depressed (uninterested, stands alone, head down, does not move with pen mates, may lie in sternal recumbency, reluctant to stand, not eating); 4 = moribund (recumbent, rarely stands, oblivious to surrounding, not eating). Clinical scores for the assessment of lameness were based on the following scale: 0 = normal gait (moves freely, no swellings); 1 = mild lameness (may have swollen joints, favours sore leg (legs) weight bearing all four legs); 2 = moderate lameness [does not weight bear when standing, walks with limp, prefers to lie, swollen joint (joints)]; 3 = severely lame (recumbent, reluctant to rise, none weight bearing, swollen joints); 4 = extreme lameness (unable to rise). Respiratory distresses were based on the following scale: 0 = normal nasal breathing; 2 = mild respiratory distress (intermittent mouth breathing, moist nose and mouth); 3 = moderate respiratory distress (mouth breathing when stressed, laboured breathing); 4 = severe respiratory distress (stands with head low, open mouth breathing, drools, tongue extended). All animals euthanized (animals restrained and administered an overdose of sodium pentobarbital by intra-venous injection) 3 weeks post *H*. *somni* bacterial challenge were subjected to a full post mortem analysis with bacteriological analysis carried out on samples of heart, lung, kidney and joints at PDS (Prairie Diagnostic Services, 52 Campus Drive, Saskatoon, SK, S7N 5B4, Canada). Additionally, animals that died or were euthanized during the trial due to extreme illness (humane intervention points) were also subjected to a full post-mortem with bacteriological analysis.

### 2.6 Enzyme-Linked Immunosorbent Assay (ELISA)

*H*. *somni* antigens described in **Table 1** were diluted in 0.05 M carbonate/bicarbonate buffer at pH 9.6 to 1 μg/ml and applied to 96-well plates (Immulon 2HB 96U: Thermo Scientific, catalog No. 3655) at 100 μl per well. Plates were covered and left overnight at 4°C. Plates were washed 6 times with reverse osmosis H_2_O and then blocked with 125 μl diluent (Tris buffered saline with 0.5% fish gelatin, Sigma G7765) for 45 minutes at room temperature. Plates were washed and serum samples (from animal trial # 2) diluted 1/100 were added, and serial four-fold dilutions were done. After two hours at room temperature, plates were washed and KPL Goat anti Bovine IgG (H+L) alkaline phosphatase labelled affinity purified antibody (Mandel catalogue No. KP-15-12-06) diluted 1/5000 was added at 100 μl/well and incubated for 1 hour. After washing, 100 μl PNPP substrate (1 g PNPP (Sigma catalog No. N3254) per 10 ml of 1% Diethanolamine (Sigma catalog No. D8885) with 0.5 mM MgCl_2_ at pH 9.8 was added for color development. The reaction was stopped by the addition of 30 μl 0.3 M EDTA per well. Plates were read at λ = 405 nm, reference λ = 490 nm on an ELISA reader (Molecular Devices SpectraMax Plus 384). Data was analyzed using microsoft excel.

### 2.7 Statistical analyses

Statistical analyses were performed using one way ANOVA, Brown-Forsythe test and Bartlett’s test for weight change. Statistical determination for sum of joints affected and sum of clinical scores was completed using Kruskal-Wallis test. Statistical determination of serum polyclonal antibodies against *H*. *somni* antigens in ELISAs was determined using Mann Whitney in GraphPad Prism 6 (http://www.graphpad.com/scientific-software/prism/). A *P* < 0.05 was considered statistically significant.

## 3. Results

### 3.1 Serological response to vaccination

**Fig 1:**
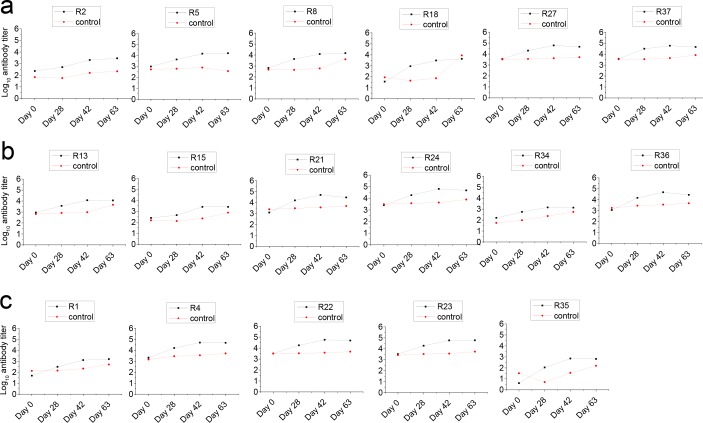
Serum antibody titers to antigens. (**1a**) Serum antibody titers to antigens in group B (R2, R5, R8, R18, R27, R37) shown in black. (**1b**) Serum polyclonal antibody titers to antigens in group C (R13, R15, R21, R24, R34, R36) shown in black. (**1c**) Serum polyclonal antibody titers to antigens in group D (R1, R4, R22, R23, R35) shown in black. Median values for serum antibody titers shown on Y axis of samples taken prior to vaccination (day 0), at the time of boost (day 28), immediately prior to *H*. *somni* bacterial challenge (day 42) and immediately prior to euthanization (day 63). “R” is denoted for rank of gene or protein. Group A, the control group, only received Emulsigen supplemented with CpG is shown in red.

Serum antibodies against *H*. *somni* antigens were determined using an ELISA procedure with individual serum samples taken prior to vaccination, at the time of boost, immediately prior to *H*. *somni* challenge, and immediately prior to euthanization. The titers against each antigen are shown in **Table 3**. Statistical determination of serum antibodies against *H*. *somni* antigens in group B or group C or group D for 21 days post challenge compared to serum antibodies in the non vaccinated group A (shown in **S2 Table**) was statistically significant at *P* < 0.05 using Mann Whitney test in a two tailed *P* value, except for antigen R18 in group B. Compared to serum samples taken prior to vaccination (day 0), median values for serum antibody titers at the time of boost (day 28) or immediately prior to *H*. *somni* bacterial challenge (day 42) or immediately prior to euthanization (day 63) against antigens in group B, C, and D increased as shown in **Table 3**. Among the antigens in group B, R27 (60688) and R37 (58009) had the highest median values for serum antibody titers at day 42 as shown in (**Table 3 and Fig 1A**). In the same manner, among the antigens in group C or group D, median values for serum antibody titers at day 42 were the highest in R21(49710), R24 (63826) and R36 (48290) or R4 (50635), R22 (57298) and R23 (57180), respectively (**Table 3 and Fig 1B and 1C**).

**Table 3:** Serological response to vaccination. Median values for serum antibody titers taken prior to vaccination (day 0), at the time of boost (day 28), immediately prior to *H*. *somni* bacterial challenge (day 42) and immediately prior to euthanization (day 63). “R” is denoted for rank of gene or protein. The vaccine for group A (placebo/control group) did not contain antigens. The vaccine for groups B, C and D contained antigens (R2, R5, R8, R18, R27, R37), (R13, R15, R21, R24, R34, R36) and (R1, R4, R22, R23, R35) respectively.

Antigen	Median values for serum antibody titers(vaccinated groups)				Median values for serum antibody titers(non-vaccinated group A)			
**Group B**	**Day 0**	**Day 28**	**Day 42**	**Day 63**	**Day 0**	**Day 28**	**Day 42**	**Day 63**
**R2**	232	491	2069	2853	69	57	161	218
**R5**	962	4272	14216	15670	525	608	772	372
**R8**	662	4228	12056	14741	460	445	602	4101
**R18**	36	913	3013	4237	87	44	72	8860
**R27**	3538	20418	60688	45566	3201	3542	4116	5118
**R37**	3675	30923	58009	44620	3433	3491	4286	8110
**Group C**								
**R13**	893	3623	11537	11129	657	812	939	4433
**R15**	252	450	2545	2512	153	131	223	759
**R21**	1228	15817	49710	29344	2397	3040	3585	4913
**R24**	2604	18543	63826	47868	3109	3724	4353	7871
**R34**	160	557	1413	1367	55	97	232	551
**R36**	1136	14571	48290	27734	1754	2876	3551	4732
**Group D**								
**R1**	49	314	1293	1573	133	143	215	506
**R4**	2106	16202	50635	47892	1472	2871	3505	5185
**R22**	3159	18044	57298	49019	3205	3361	3789	4727
**R23**	3291	18895	57180	58139	2621	3296	3606	5381
**R35**	4	109	710	647	32	5	35	154

### 3.2 Response to infection with *H*. *somni*

**Fig 2:**
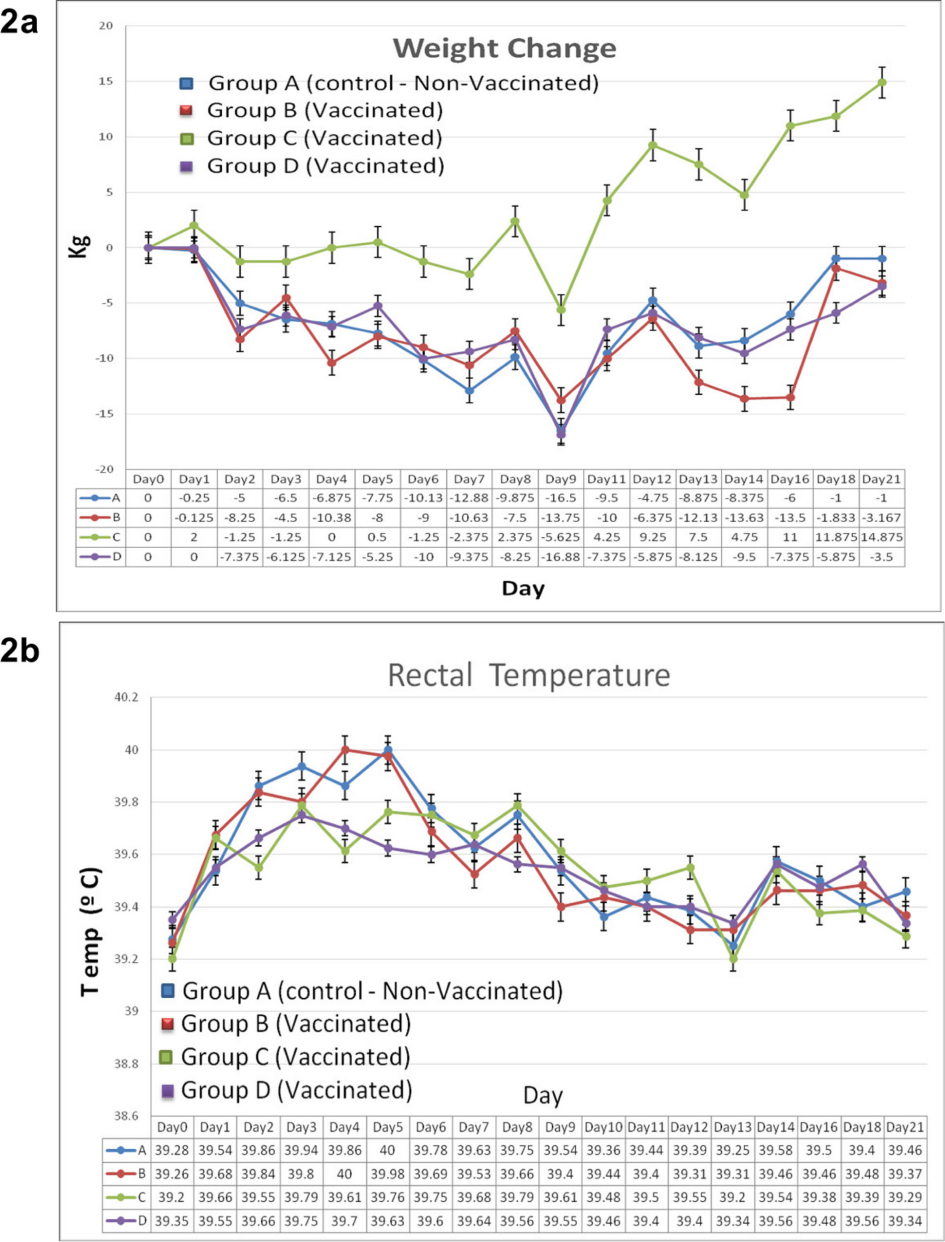
Weight and temperature change in response to infection with *H*. *somni*. (**2a**) Weight change among group A (non vaccinated/ placebo group) and groups B, C and D (vaccinated) animals measured for 21 days post challenge. (**2b**) Rectal temperature among group A (non vaccinated/placebo group) and groups B, C and D (vaccinated) animals measured for 21 days post challenge. Animal groups are shown in colors: group A (blue), group B (red), group C (green) and group D (purple). Standard error bars included.

The response to infection with *H*. *somni* on body weight of animals in all four groups (A, B, C, D) is shown in **Fig 2A**. After the *H*. *somni* challenge all animals were monitored on a daily basis for change in body weight. The initial loss/gain of body weight was observed at one day post *H*. *somni* challenge. One animal in the control group (A) and two animals in the vaccinated group (B) were euthanized on day 17 post challenge due to being severely ill. Statistical determination of weight change for 21 days post challenge that included the three euthanized animals between vaccinated groups B, C, D and control group A was not statistically significant at *P* < 0.05 using one way ANOVA (*P* value 0.3798), Brown-Forsythe test (*P* value 0.4931) and Bartlett’s test (*P* value 0.5045) in GraphPad Prism 6. In the same manner, statistical determinations of weight change for 16 days post challenge which included all animals between vaccinated groups B, C, D and control group A, was not statistically significant at *P* < 0.05 using one way ANOVA (*P* value 0.2661, Brown-Forsythe test (*P* value 0.5510) and Bartlett’s test (*P* value 0.4449). Finally, even though there was no significant difference in weight loss/gain between any groups and the control, group C (**Fig 2A**) continued to gain weight following infection. The response to infection with *H*. *somni* on rectal temperature of animals in all four groups (A, B, C, D) is shown in **Fig 2B**. As shown in **Fig 2B**, there was no difference in rectal temperature between vaccinated groups B, C, D and control group A.

**Fig 3:**
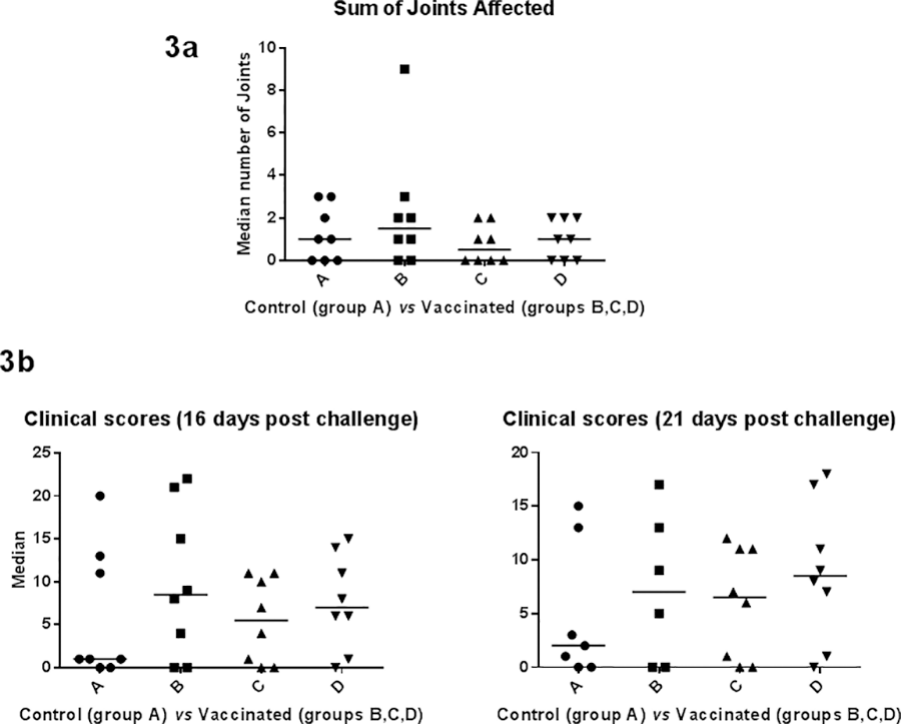
Affected joints and clinical symptoms in response to infection with *H*. *somni*. (**3a)** Sum of joint lesions among control group A *vs* vaccinated groups B, C and D animals measured from day 1 to 21 post challenge. (**3b**) Sum of clinical scores measured from day 1 to 16 post challenge or day 1 to 21 days post challenge. Two animals in group (B) were euthanized on day 17 post challenge due to being severely ill.

The response to infection with *H*. *somni* on the sum of joint lesions is shown in **Fig 3A**. All animals euthanized were subjected to a full post mortem analysis with bacteriological analysis carried out on joint samples. Statistical determination of the sum of joint lesions for 21 days post challenge that included the three euthanized animals between vaccinated groups B, C, D and control group A was not significant at *P* < 0.05 using Kruskal-Wallis test (*P* value 0.5814) in GraphPad Prism 6. Group C had the lowest sum of joint lesions (six) among the three vaccinated groups (B, C, and D) as well as compared to the control group A (**S3 Table)**.

The response to infection with *H*. *somni* on the sum of clinical scores is shown in **Fig 3B**. All animals were subjected to a full post mortem analysis with bacteriological analysis carried out on samples of heart, lung, kidney and joints (**S4 Table**). Statistical determination of the sum of clinical scores for 16 days post challenge was not statistically significant at *P* < 0.05 using Kruskal-Wallis test (*P* value 0.7152). Statistical determination of the sum of clinical scores for 21 days post challenge that included the three euthanized animals was not statistically significant at *P* < 0.05 using Kruskal-Wallis test (*P* value 0.7140) in GraphPad Prism 6. The total sum of clinical scores for 21 days post challenge for groups A, B, C, and D were 54, 87, 48 and 71 respectively (including the three euthanized animals). Even though there was no significant difference in clinical scores, group C had the lowest sum of clinical scores of 48 among the three vaccinated groups (B, C, D) as well as compared to the control group A.

## Discussion

*H*. *somni* is an economically important global pathogen responsible for significant economic losses to the livestock industry not only in North America but globally (e.g. Paraná State of southern Brazil, Hungary, UK, Argentina, Nigeria, South Africa) [3,43,44,45,46,47,48]. Vaccination has proven to be the most efficient method of protecting humans and animals from infectious diseases and is also one of the most cost effective interventions in preventing epidemics [28]. Current vaccines against *H*. *somni* have shown to be of limited efficacy where feedlots, ranchers and cattle producers practising proper BRD management protocols still face considerable animal losses due to systemic infections [5,22,23,49,50]. Genome-based reverse vaccinology has the advantage of identifying a larger number of vaccine candidates in a relatively short period of time [25–28]. Using this strategy we were able to test the efficacy of a subset of proteins identified in a field isolate of *H*. *somni* (e.g. strain AVI1) isolated in 2012. Moreover, proteins localized on the cell surface (e.g. OMPs) and also conserved between strains may contribute to bacterial virulence and host immunity [4,24]. Multiple component vaccines have the added advantage of containing more than one antigen which may increase efficacy [5].

Vaccines containing a single OMP antigen or surface fibrillar network, immunoglobulin binding protein A (IbpA) have been previously assessed by other groups [5,18]. For example, lipoproteins p40 (40 kDa protein) and p31 (31 kDa protein: homologous to lipoprotein Plp4 of *Mannheimia haemolytica* A1) when used as vaccine candidates exert a protective effect in mice against *H*. *somni* related septicemia [5]. Calves vaccinated with a domain of the IbpA annotated as DR2 were also protected from *H*. *somni* related pneumonia [18]. In our study, vaccination with antigens in group B (R2, R5, R8, R18, R27, R37), group C (R13, R15, R21, R24, R34, R36) or group D (R1, R4, R22, R23, R35) using a large animal model (bovine) followed by *H*. *somni* bacterial challenge provided the added advantage of screening combinations of recombinant proteins (NCBI Blast comparison of antigens is shown in **Table 1**). The results in trial # 2 demonstrate that animals vaccinated with the combination of antigens in group C had the lowest number of clinical signs (**Fig 3B**), joint lesions (**S3 Table**), overall illness (**S4 Table**) and the highest gain in weight (**Fig 2A**) compared to the control group, but none of which was significantly different from the control group. Additionally, group C had the lowest number of clinical signs, joint lesions, overall illness and the greatest weight gain among the other vaccinated groups (B or D). Statistical determination of serum antibodies against *H*. *somni* antigens in group C compared to sera from the control group was significant. The prospect of using conserved OMPs as subunit vaccines is ideal for Gram-negative bacteria that show highly variable sequence diversity [5]. The results presented in trial # 2 show that the combination of R13, R15, R21, R24, R34 and R36 [blastp on NCBI, R13: *H*. *somni* porin, WP_012340590.1 (89%); R15: LPS assembly protein LptD, WP_012341555.1 (99%); R21: *H*. *somni* membrane protein, WP_011609419.1 (93%); R24: OMP1, ACA32123.1 (99%); two hypothetical proteins, ABI25169.1 (99%); WP_011608601.1 (99%)] provided protection against systemic infection by *H*. *somni* challenge. However, their ability to protect against the pneumonic form of the disease was not tested in this study. Among these six antigens, the genes coding for R21, R24 and R36 were fused to the *M*. *haemolytica lktA* gene. The leukotoxin from *M*. *haemolytica* has been shown to be protective against *M*. *haemolytica* infections, but it is not cross protective against other pathogens [51]. If protection against *H*. *somni* challenge was due to the leukotoxin portion of the fusion proteins, then other antigens such as R27, R37 in group B or R4, R22, R23D in group D that were also fused to leukotoxin, should have produced a similar effect. Nonetheless, titers of serum antibodies in leukotoxin fused proteins (R21, R24, R36) among the group C antigens on day 42 was above 40,000 compared to R15 and R34 (histidine tagged proteins) and R13 (inclusion body preparation) (**Table 3**). Similarly, leukotoxin fused antigens, R27 and R37 in group B and R4, R22 and R23 in group D had serum antibody titers above 50,000 on day 42 (**Table 3**). The results from this study may indicate that there is a close correlation between the antibody response and leukotoxin fused antigens. Hypothetical proteins such as R34 (NCBI accession No. ABI25169.1) or R36 (NCBI accession No. WP_011608601.1) in group C are interesting in that their biological function is unknown and may have made a significant contribution to the total protective effect exerted by the multi-component vaccine. Therefore, future structural studies of these two proteins will be necessary to determine their immunogenic properties.

The antigens in group C may not all be needed to induce protection. Future animal trials using group C antigens with animal groups receiving each antigen alone or combinations of two-to-three antigens should be tested. This may result in better protection against histophilosis in cattle. In conclusion, the subunit vaccine used in group C exhibits a trend towards protective immunity in cattle and would be a good candidate for further analysis to determine which proteins were responsible for the trend towards protection.

## Supporting Information

S1 Table DNA sequences and amino acid sequences of antigens used for vaccine groups B, C, and D, from *H*. *somni* strain (AVI1) used in the animal trial #2 at VIDO-Intervac, Saskatoon. “R” is denoted for rank of gene or protein.
(DOCX)

S2 Table Statistical determination of serum antibodies using Mann Whitney test “R” is denoted for rank of gene or protein. The vaccine for group A (placebo/control group) did not contain antigens. The vaccine for groups B, C and D contained antigens (R2, R5, R8, R18, R27, R37), (R13, R15, R21, R24, R34, R36) and (R1, R4, R22, R23, R35) respectively.
(DOCX)

S3 Table The sum of joint lesions for each animal measured from day 1 to 21 post challenge for all groups: A (non-vaccinated) and B, C, D (vaccinated). (DOCX)

S4 Table Full post-mortem analysis of euthanized animals. Bacteriological analysis carried out on samples of heart, lung, kidney and joints. Positive cultures for *H*. *somni* are shown in dark green.
(DOCX)

## Abbreviations

OMPs: outer membrane proteins
ORFs: open reading frames
