# Supplementary material for: Cattle Immunized with a Recombinant Subunit Vaccine Formulation Exhibits a Trend towards Protection against Histophilus somni Bacterial Challenge
Source: PLoS One. 2016 Aug 8;11(8):e0159070. doi: 10.1371/journal.pone.0159070 (PMC4976985; doi:10.1371/journal.pone.0159070)
Supplement: S1 Table — “R” is denoted for rank of gene or protein. (DOCX) [file pone.0159070.s001.docx]

**S1 Table.** DNA sequences and amino acid sequences of antigens used for vaccine groups B, C, and D, from *H. somni* strain (AVI1) used in the animal trial #2 at VIDO-Intervac, Saskatoon.

| **Antigen** | **DNA sequence from *H. somni* strain (AVI1)** | **Amino acid sequence from *H. somni* strain (AVI1)** |
| --- | --- | --- |
| R2 ( group B) | AGCATGACCCATCCGTCTTTTACTCACACGGAATGAAAAAAAATGTCTACAAAACCTTTG  TTTAAACTTAAGCCAATAACATTGGCTGTCAGCACGATTTTTTTACCTTTTACTGAGGCG  GTTGCCGATACTGAATCACCGAGTAGCAATACAGAAGCAGTGCTGGAGTTAGAAGCTATC  CAGGTGCAAGCCAAACACGAGATCAGCAGACATGACAATGAAGTCACTGGTTTGGGTAAG  GTGGTCAAAAGCAGTGAAGACATTGATAAAGAACTGATTTTGAATATTCGCGATTTGACC  CGTTATGATCCCGGTATTTCGGTGGTGGAGCAGGGACGTGGTGCAACGTCAGGCTATGCA  ATGCGTGGTGTTGACAGAAACCGCGTGGCTATGTTGGTGGACGGCTTGGGACAGGCACAG  TCGTATTCTACCTTGAAATCCGATGCCAATGGCGGGGCGATTAATGAAATTGAATATGAG  AATATTAAGTCAATTGAATTGAGCAAGGGATCCAGTTCGGCAGAATACGGTAGCGGTGCC  TTGGGCGGTGCTGTAGGGTTTCGTACCAAAGAAGCTGATGATGTGATTAAAGAGGGGCAA  AACTGGGGCTTGAACAGTAAAACGGCTTACAGCAGCAAAAACAGCCAGTTTACCCAATCC  GTTGCCGGTGCGTTCCGTGTCGGCGGTTTTGACAGTTTGGCGATTTTTACCCATCGTAAA  GGTAAGGAAACCCGCGTGCATCCTGCCGCCGAAGAAATACAACATACTTACCAACCATTG  GAAGGGTATTTTAATCGGTATGATGTTGACCAAAGAAACGGAACGCCTGTTCGGGCGAAT  GCGTATTATATACTTGCCGATGAATGCTCTAATCTAAGTGATCCGAATTGTCGTCATGCT  AAGGCCAAGACGAATTTGTCGGGGGTGCTGGAGAACAATCCTAATTGGACGTCCGAAGAG  CAGGCGCAGGCTGCTAAAATGCCGTATCCGACACGTACCGCCTCTGCCAAAGATTATACG  GGTCCTGACCGCATCAGCCCTAATCCGATGGACTACCAAAGTCGCTCTTTCTTCTGGAAA  GGTGGTTACCGCTTGTCGCCTAACCATTATGTCGGCGGGGTGTTGGAGCATACGAAGCAG  CGTTACGATATCCGTGATATGACGCAACGGGCGTATTACACGAAAGAGGATATCTGCCGC  AGCGGATCCAGTTGCCAAACGTTGGATAAAAACGATACGGACAAAGGTAATTTCGGTATC  ACGTTGACTGATAATCCTTTGGACGGTTTGGTATATGATGCCGGCAATCAAGCGCGTGGC  GTGCGGTACGGACGGGGTAAGTTTTTTGATGAACGCCATACGAAAAATCGCTCGGGTATT  TTTTACCGCTATGAGAATCCCGATAAAAATTCTTGGGCAGATAGCTTGACCTTGAGTATT  GACCGCCAAGATCTCAAACTATCGAGCCGTATCCATTGGACGTATTGCACCGATTATCCT  CATGTGGCACGTTGCCGTGCCAGCTTGGACAAACCTTGGTCTAATTACCGTACCGAGAAA  AACGATTATCAAGAACGACTCAATTTGGGACAATTCAATTGGGAAAAAACTTTTAATCTG  GGCTTTACCACGCATAAGGTGAATATCGCCGCCGGCTTTGGTACACATCGCTCCACCTTA  CAACATGGCGACTTATATGCTGAATATGTCACCTTGCCACCGTATACAGAGGAAAAAGTGTATGGCGATGATAATAAGGTCAAACAAAATCCGACACCAGAAGAAAAAGAGAAATTACAA  TACGGCAATGGTTCTTATAACAAACCTCGCGTATATAGACGTAAAAACACGCCGGAATTA  AAAACTGTCAATGGGTGCAATGAGACAGCAGGTGACAATCGCGACTGCACGCCACGTGTG  ATTACGGGCAGACAGTATTACCTTGCCTTGCGTAACCATATTGCCTTTGGTGAATGGGCA  GACTTGGGGTTGGGCGTGCGGTACGACAACCATACTTTCCGCTCGACTGACCCGTGGACC  AAAGGTGGCAACTACCACAACTGGTCGTGGAATGCGGGCGTGAGCCTCAAACCGACCCGC  CACTTTGTCGTGTCTTACCGTGTGTCCAGCGGTTTCCGTGTCCCCGCTTTTTATGAGCTG  TACGGCGTGCGTACGGGGGCTTCTGGTAAAGACAATCCACTCACACAAAAAGAGTTCTTG  AGCCGTAAACCGTTGAAAAGCGAAAAAGCCTTTAACCAAGAAATTGGTTTGGCCGTTCAG  GGAGATTTTGGTGTGATAGAGACCAGTTTCTTCCAAAACAATTATAAAAATCTGCTTGCC  CGTGCAGATAAACATGTCGAGGGATTGGGTTATGTAACCGATTTTTACAACACCCAAGAT  GTCAAACTCAACGGTATCAATATCTTGGGTAGAATCTACTGGGCAGGCATCAGCGATAGG  CTGCCTGAAGGCTTGTATTCCACACTTGCTTACAACCGTATCAATATCAAAGCACGCAAA  TTGCACGACAATTTTACCAATGTGTCTGAGCCGACATTGGAAGCCGTGCAACCGGGACGC  ATTATTGCAAGTATCGGCTATGATGACCCTGAGGGCAGATGGGGCCTTAATTTAAGCGGC  ACCTACTCTCAAGCCAAACAACGTGACGAAGTGGTCGGCGAAAAAGTGTTCGGCAAGGGT  GGCAGCATTAAACGGACGATCAACAGCAAACGCACTCGTGCTTGGTATATTTATGATTTG  ACGGCATACTACACTTGGAAAGAAAAATTCACGTTGAGAGCTGGTATCTATAATTTAACC  AATCGTAAATATAGCACATGGGAAAGTGTGCGTCAGTCCTCTGCCAATGCGGTCAATCAA  GACCTAGGTACACGTTCGGCACGTTTTGCCGCACCGGGCAGAAACTTTACCGTGAGTATG  GAAATGAAGTTTTAATTAAAAAACTGTCTGCAAGCTGTTTAAAAAACAGTTAAGATG | MSTKPLFKLKPITLAVSTIFLPFTEAVADTESPSSNTEAVLELEAIQVQAKHEISRHDNEVTGLGKVVKSSEDIDKELILNIRDLTRYDPGISVVEQGRGATSGYAMRGVDRNRVAMLVDGLGQAQSYSTLKSDANGGAINEIEYENIKSIELSKGSSSAEYGSGALGGAVGFRTKEADDVIKEGQNWGLNSKTAYSSKNSQFTQSVAGAFRVGGFDSLAIFTHRKGKETRVHPAAEEIQHTYQPLEGYFNRYDVDQRNGTPVRANAYYILADECSNLSDPNCRHAKAKTNLSGVLENNPNWTSEEQAQAAKMPYPTRTASAKDYTGPDRISPNPMDYQSRSFFWKGGYRLSPNHYVGGVLEHTKQRYDIRDMTQRAYYTKEDICRSGSSCQTLDKNDTDKGNFGITLTDNPLDGLVYDAGNQARGVRYGRGKFFDERHTKNRSGIFYRYENPDKNSWADSLTLSIDRQDLKLSSRIHWTYCTDYPHVARCRASLDKPWSNYRTEKNDYQERLNLGQFNWEKTFNLGFTTHKVNIAAGFGTHRSTLQHGDLYAEYVTLPPYTEEKVYGDDNKVKQNPTPEEKEKLQYGNGSYNKPRVYRRKNTPELKTVNGCNETAGDNRDCTPRVITGRQYYLALRNHIAFGEWADLGLGVRYDNHTFRSTDPWTKGGNYHNWSWNAGVSLKPTRHFVVSYRVSSGFRVPAFYELYGVRTGASGKDNPLTQKEFLSRKPLKSEKAFNQEIGLAVQGDFGVIETSFFQNNYKNLLARADKHVEGLGYVTDFYNTQDVKLNGINILGRIYWAGISDRLPEGLYSTLAYNRINIKARKLHDNFTNVSEPTLEAVQPGRIIASIGYDDPEGRWGLNLSGTYSQAKQRDEVVGEKVFGKGGSIKRTINSKRTRAWYIYDLTAYYTWKEKFTLRAGIYNLTNRKYSTWESVRQSSANAVNQDLGTRSARFAAPGRNFTVSMEMKF |
| R5 (group B) | ATGGCTACTGTTAACAGAGGATCGCATCACCATCACCATCACAACAGATCTATGAAAAAA  ACAATTATTGCATTATCTATCGCAGGTTTTGTTGCTTCTGTACAAGCGGCACCTCAAGCA  AATACGTTCTATGCCGGTGCGAAAGCCGGTTGGGCATCTTTCCATGACGGTCTTAATCAA  TTTGAAAATTCTGCAGAAAAGAAAGGAACAGTTCGTAATTCAGTAGCTTATGGTATTTTT  GGTGGTTACCAAATTACCGATCATGTTGCTGTAGAGTTGGGTTCTGAGTATTTCGGTCAA  GCAAAAGGTCGTAAAGAGAAAAAAGAAGCTAAACATACCGCTCAAGGGATGCAATTAGGC  TTAAAAGCAAGCTACCCTGTATTAGAAGGCTTGGATATTTACGGTCGTGTAGGTGCGGCG  TTAATTCGTTCTAATTATGTTGATGTTAAACATTTAGAAAGTGATAAAGATGTAAAAAAC  ACTTTAAAAGTTTCTCCGGTTTTTGCGGCGGGTGTTGAATACAGCCTACCTTCTTTACCG  GAATTGGCATTACGTTTGGAATATCAATGGGTTAAAGGCGTTGGTAAAGCACGTAAGAAA  GACAGTGGTGAACGCTTAGACTATACACCAAGTATCGGTGCAGTAACACTTGGCTTATCT  TACCGTTTCGGTCAAAAACCGGTTATGGCACCTGAAGTAGTAAACAAAGTGTTCAGCTTA  AATTCAGATGTGAATTTTGCCTTCGCTAAAGATACATTAAAACCTGAAGCTCAACAAACA  TTGGACGGTGTTTATGGTGAAATCGCACAATTAAAAACCGCACAAGTTTCTGTTGCCGGT  TATACAGACCGTATCGGTTCTGATGCGTCAAACTTAAAATTATCACAACGTCGTGCGGAT  ACTGTAGCAAATTATTTAGTCTCTAAAGGTGTTGCTCAAGATGCCATTAGTGCGGTTGGT  TACGGTGAAGCAAATCCGGTAACCGGTATGAAATGTGATGCGGTTAAAGGTCGCAAAGCA  TTAATCGCATGTTTGGCAGAAGATCGTCGTGTTGAAATCTCAGTGAAAGGTAGCAAATAA | M A T V N R G S H H H H H H N R S M K K T I I A L S I A G F V A S V Q A A P Q A N T F Y A G A K A G W A S F H D G L N Q F E N S A E K K G T V R N S V A Y G I F G G Y Q I T D H V A V E L G S E Y F G Q A K G R K E K K E A K H T A Q G M Q L G L K A S Y P V L E G L D I Y G R V G A A L I R S N Y V D V K H L E S D K D V K N T L K V S P V F A A G V E Y S L P S L P E L A L R L E Y Q W V K G V G K A R K K D S G E R L D Y T P S I G A V T L G L S Y R F G Q K P V M A P E V V N K V F S L N S D V N F A F A K D T L K P E A Q Q T L D G V Y G E I A Q L K T A Q V S V A G Y T D R I G S D A S N L K L S Q R R A D T V A N Y L V S K G V A Q D A I S A V G Y G E A N P V T G M K C D A V K G R K A L I A C L A E D R R V E I S V K G S K |
| R8 (group B) | ATGAAAAAACTGTTAATTGCAAGCCTATTATTTGGCTCGACTGGTCTGGTTGCCGCACCG  TTTGTCGTGCAAGATATTCGTATAAACGGTATTCAAGCAGGTAAGGAAAGTGCGGTGTTA  TCTGGATTGCCGGTTCGAATTGGACAAAAAGCAACGGAGAGTGATATTTCAAATGTCGTA  AGACTATTATTTTTGCGTGGCTATGATAATGTGCAAGCTGTTCGTGAAGGAAATACCTTA  GTCATTTCTGTAGTACCACGCTTAGTGATTGCGGAAGTGAAAGTGGAGGGAAATGAATCT  ATTCCTAGTGAAGCAATACGAGAAAACTTGAAAGCGAACGGATTTGCTAGCGGTGATATT  TTAAATCGAGAAAAATTGGAAGCGTTTCGTGGAAGTTTAGTTGATCATTATCAGTCTGTC  GGTCGTTATAATACGACTGTAGAGGCAATTGTTAATCCATTATCAAATGATCGGGCAGAA  GTAAAACTTAAAATAAAAGAAAGTGATGTGGCAAAACTGAAAGAAGTGCGTTTTGAGGGC  AATGAAGCATTTAGCAGTAGCCAATTACAAGAGCGGATGGAATTACAGCCGGATGCTTGG  TGGAAGTTATTTGGGAATAAATTTGATACCAATCAATTTAACCAAGATTTGGATCTTATT  CGTGATTTTTATTTAGAACATGGTTATGCAAAATTTCAAATCGTTGGTACCGATGTTCAG  CTAAATGACGAAAAAACTGAGGTTAGAGTTAGAATTCAAGTTAACGAAGGTGATAAGTAT  TCTGTAAATAGTGTCCGAATCGTTGGTGATGTAGGCGGTATGTCAGAAGAGTTAGCACCG  TTACTTAAAAACATTTATGTCGGAGAAACTTTCCGTCGTAGTGAAGTTAGCAGTGTTGAA  CAATTGATAAAAGCAAAATTAAGCGAGCAAGGCTATGCAACGGCAAAAGTAGATGTTAGT  TCAGTCTTTAATGAAGAGAACAAAACGATTGATTTAACGTTTGTTGTTGATGCGGGACAT  CGTTATTCTGTTCGTCAAATCCGCTTTGAGGGGAATACGATTACTGCCGATAGCACATTA  CGTCAAGAAATGCGTCAACAAGAGGGGGCTTGGTTATCATCTCAATTAGTTGAGTTAGGG  AAAATTCGTCTAGAGCGTACCGGATTTTTTGAGTCAGTGGAAACGGAAACTCAAACCAAT  CAACAAATTAACGACCAAGTTGATGTCATTTATCGAGTCAAAGAACGCAATACAGGGAGT  TTGAACTTCGGTATTGGTTATGGTACCGAAAGTGGTATAAGTTATCAGGCAAGTATTAAA  CAGGATAATTTCTTGGGCATGGGTTCCTCGATTAGTTTAGCCGGTTCACGCAACAATTAT  GGCACAAGTCTTAATTTAGGTTATAACGAACCGTACTTTACTAAAGATGGCGTAAGTTTA  GGTGGAAACGCATTTTTTGAAAAACACGATAACTCAAAAAGTGATACTGCAGCCGCCTAT  GGACGTACAACTTACGGTCTAAGTGGAAATTTAAGTTTTCCGGTCAATGAAAATAACTCC  TATTATTTAAGTTTAGGGCATATTTATAGCCAATTAAAAAATGTGACGAAAGAATATAAT  CGTGATTTATATCGCAAATCAATGGGGTATCCTGATTCTAATAGATGGAATTTTAAATCG  CATGACTTTGAATTTTCATTCGGCTGGAACTATAACAGCTTAAACCGTGGCTATTTGCCT  ACCTCCGGAGTAAGAGCGAATGTTGGCGGTAAAGTAACAATCCCAGGTTCAGATAACAGA  TACTATAAAGTGAGTGCTGAAGTGCAGGGTTTTTATCCATTGGATCGTGATCATTATTGG  GTACTGACAGGTAGATTGTCAGGATCATATGCAAACGGTTTTGGCGGTAAACGTTTACCG  TTTTATCAAACTTACTCTGCTGGAGGTATAGGAACTGTACGTGGTTTTGAATATGGTGCA  ATAGGACCAAAAGCGATTTATCAGACTGGTTGTAGTAATGGTGCTAGTTCTACATCAACC  ACAAGCAACAACAATATGTATAAATGCCATAGCAAAGATATTGTTGGCGGAAATGCCATG  ACATTGGCAAGTATTGAACTTATTGTTCCGACGCCGTTTGTAGCAGAGAAAAACCAACGA  TCAGTGAGAACCTCTATCTTTGCCGATGCTGGTTCTGTATGGAATACTAAATGGAAATCA  GATGGTAAACCATTTGCTGGAGATAAAAATATACCTGACTACGGGAACCCAGCTCGTATA  AGAGTTTCAGCAGGGATTGCGTTCCAATGGTATTCTCCGATTGGACCTTTAGTATTCTCT  TATGCTAAACCACTGAAAAAATATCAAGGTGACCAAATTGAACAATTCCAATTTAGTATT  GGTGGTTCTTTTTAG | MKKLLIASLLFGSTGLVAAPFVVQDIRINGIQAGKESAVLSGLPVRIGQKATESDISNVVRLLFLRGYDNVQAVREGNTLVISVVPRLVIAEVKVEGNESIPSEAIRENLKANGFASGDILNREKLEAFRGSLVDHYQSVGRYNTTVEAIVNPLSNDRAEVKLKIKESDVAKLKEVRFEGNEAFSSSQLQERMELQPDAWWKLFGNKFDTNQFNQDLDLIRDFYLEHGYAKFQIVGTDVQLNDEKTEVRVRIQVNEGDKYSVNSVRIVGDVGGMSEELAPLLKNIYVGETFRRSEVSSVEQLIKAKLSEQGYATAKVDVSSVFNEENKTIDLTFVVDAGHRYSVRQIRFEGNTITADSTLRQEMRQQEGAWLSSQLVELGKIRLERTGFFESVETETQTNQQINDQVDVIYRVKERNTGSLNFGIGYGTESGISYQASIKQDNFLGMGSSISLAGSRNNYGTSLNLGYNEPYFTKDGVSLGGNAFFEKHDNSKSDTAAAYGRTTYGLSGNLSFPVNENNSYYLSLGHIYSQLKNVTKEYNRDLYRKSMGYPDSNRWNFKSHDFEFSFGWNYNSLNRGYLPTSGVRANVGGKVTIPGSDNRYYKVSAEVQGFYPLDRDHYWVLTGRLSGSYANGFGGKRLPFYQTYSAGGIGTVRGFEYGAIGPKAIYQTGCSNGASSTSTTSNNNMYKCHSKDIVGGNAMTLASIELIVPTPFVAEKNQRSVRTSIFADAGSVWNTKWKSDGKPFAGDKNIPDYGNPARIRVSAGIAFQWYSPIGPLVFSYAKPLKKYQGDQIEQFQFSIGGSF |
| R18 (group B) | ATGAAACTATCACGTTTTGTATTATCTGCTGTTGTAGCAGCAACATTGTCGGCTTGTGGT  AATTTAAGTAATGTCACCGAAGAGGGTACATCAGATAACTTGAAGTGGCCTAAAATTGAT  CAGTCAAGATTTAACCATGATGGTAGCCAATTTGGATCGTGGCCAAACTGGGATAACCTA  CGTATGGTTGAACGTGGAATGAATAAAGATCAACTGTATAACCTATTGGGACGTCCGCAT  TTTTCGGAAGGTTTATATGGTGTTCGTGAGTGGGACTATGCTTTTAATTATCGTGAGAAT  GGCGAGCATAAAATTTGCCAATACAAAATTTTATTCGATAAAAATATGAATGCCCAAAAT  TTCTATTGGTTTCCAAATGGTTGTAACGGTTATGCATCTTTTAGTTTAAATACGGATCTC  TTATTTGATTTTGATCAAGATACGTTGACCTCAAAAGGTGAAGAAGCTGTTGATAATGTT  GCAATGCAATTAGAAGCGTTTTCAGCTAAAGAAATTAAAATTGTTGGTTTTACTGATCGA  TTGGGTACAGATTCTTATAACTTGGATCTTTCTCAACGCCGAGCTGATCGTGTTAAAGAG  CGTTTAATTGAAAAAGGGTTAAATATTGATATTATCGCTATTGGATATGGTGAAACGCAA  CAGATTAAGGCTTGCAATGATGTGCCAGCTAAAGAACTAAAAGATTGCTTACGTCCAAAC  CGTCGTGTTGAAATTTCAGCATACGGAAACATCTCAAAAAAATATGGTAATGGTGAGCTG  AAGGGGGGTACAACTGGTCCATCATTATACTACGAAAAATAA | MKLSRFVLSAVVAATLSACGNLSNVTEEGTSDNLKWPKIDQSRFNHDGSQFGSWPNWDNLRMVERGMNKDQLYNLLGRPHFSEGLYGVREWDYAFNYRENGEHKICQYKILFDKNMNAQNFYWFPNGCNGYASFSLNTDLLFDFDQDTLTSKGEEAVDNVAMQLEAFSAKEIKIVGFTDRLGTDSYNLDLSQRRADRVKERLIEKGLNIDIIAIGYGETQQIKACNDVPAKELKDCLRPNRRVEISAYGNISKKYGNGELKGGTTGPSLYYEK |
| R27 (group B) | ATGAAAAAAATGACTTTATCATTAGCTATTTTACTTAGTTTGGGATTAGCTGGGTGTGCC  AATGAAGATATATACAGTGGTAATGTATATAGTGGCACACAAGCTAAATCAGCTCGTTCA  ATCAGTTATGGTATCATCGAATCAATCCGTCCGGTTAAAATTCAAGGCGATAACCAAGGT  GTGATTGGGACTGTTGGTGGTGGTGCTTTAGGTGGAATAGTGGGATCTAGTATAGGCGGT  GGTACCGGACAGGCAGTCGCTGCAGCAGTTGGTGCTATTGCCGGAGCGGTAATTGGCAGT  AAAGTAGAAGAAAAAGCAAGTCAGATTAATGCGTTGGAACTTGTTATCAGAAAAGACAAT  GGACAAGAAATTGTTGTTGTGCAAAAGTATGATGCAACTTTAGTGCCGGGAGCTCGAGTT  CGTATTGTTGGTGGCTCCACTTTAAATGTTTCTGTAATGTAGCTCTGCTATTATGGTTAT  TATATAGAG | MKKMTLSLAILLSLGLAGCANEDIYSGNVYSGTQAKSARSISYGIIESIRPVKIQGDNQGVIGTVGGGALGGIVGSSIGGGTGQAVAAAVGAIAGAVIGSKVEEKASQINALELVIRKDNGQEIVVVQKYDATLVPGARVRIVGGSTLNVSVM |
| R37 (group B) | ATGGCTCAAGCTCAACAATCAAATAGTAGTAATATTGATGTTAGTAATGGGCATGTTTAT  ATTGGTGAAGTATCCCAAAAAGTTACCGGATTGAACCGTGGTACTGAAGACAAAGCCGTT  GCTATTGGAAAAGGCTCAAAAGTCGGCGAGTCCGCTGTTGCTGTAGGTTATGAGGCAGAT  GCTCATCTCGAAGGGGCTACTGCAGTAGGGCGTGGAACTAAAACTCAAGCTTATGCCGTG  GCAATGGGCTACCAAGCAAATGCAGGTGTACAGGCGGTTTCTATAGGTAACAAATCCAAC  GCACAAGGAAAGTTTGCAGTAGCAGTAGGTGATGAGGCAAACGCAACTGCGAAATTTGCT  TCGGCTCTTGGACAAGGTGCTACGGCTTCTGGCAATTATAGTAATGCTTTTGGTTATAAA  TCTAAAAGCATCAATGAATTATCTACAGCAATAGGTTGGGATTCACATGCTGCTGGACTT  AGATCACAAGCCATCGGTCCTCAAGCAAAAGTGTATGGAGATCGCTCTTTAAGTATTGGT  AACGATGTACAAACACATAAAGAACGCTCATTCTCAATTGGTAGTAGTATTGTAAACAAA  GGAACTAAAACTACCGTTATCGGTAATAATATTAATGTTGAGGTTGATAACTCCGTCTTC  CTCGGTGATTCTTCTGCTTATGTAGAAAAAGGCGAAACAACGGGCGGTATCGGTAAAGTC  AACGGCAATTACGCCGGCGTTGATGCAAAAGGCGTCGTTTCCGTGGGCAGCAAAGGCAAT  GAACGCCGTATTCAAAATGTTGCCGCCGGTTTGCTTTCTCATCAATCAACCGATGCCGTC  AACGGAAGCCAATTACACGCCACTAACCAACGTCTTGAAGAAGTCAACAAAGACGCAAAA  GCCGGTATCGCCGCCGCGATGGCTTTTAAAGACGTGCCTTTCGTCCCGGGTAAATGGTCT  TATGCCGCCGGTGCCGCTCATTATAGCAGCGAAAGTGCGGTCTCTTTAAACCTCGGCAGA  ACTTCCAATGATGGTAAATGGGCTGTCTCCGGCGGTATGTCCTCCGACAGCCGTGGTCGC  CTCGGTTTCCGTGTCGGCGTCAGCGGTGTGTTTAACTAA | MAQAQQSNSSNIDVSNGHVYIGEVSQKVTGLNRGTEDKAVAIGKGSKVGESAVAVGYEADAHLEGATAVGRGTKTQAYAVAMGYQANAGVQAVSIGNKSNAQGKFAVAVGDEANATAKFASALGQGATASGNYSNAFGYKSKSINELSTAIGWDSHAAGLRSQAIGPQAKVYGDRSLSIGNDVQTHKERSFSIGSSIVNKGTKTTVIGNNINVEVDNSVFLGDSSAYVEKGETTGGIGKVNGNYAGVDAKGVVSVGSKGNERRIQNVAAGLLSHQSTDAVNGSQLHATNQRLEEVNKDAKAGIAAAMAFKDVPFVPGKWSYAAGAAHYSSESAVSLNLGRTSNDGKWAVSGGMSSDSRGRLGFRVGVSGVFN |
| R13 (group C) | ATGAAAAAGACATTAGTAGCATTAGCGGTAGCGGCAGTTGCGGCATCAGCAAACGCAACA  ACTGTTTATAATCAAAACGGTACCAAAGTAGAAGTTGGCGGTCGTGTTGATGTAATGTTA  GGTAAATTCGGTGATGCTCAACGTACCGATTTACGTAATAACGGATCTCGTGTGGAATTT  AAAGCGGAGCATGAAGTACAAAACGGCTTAAAAGCAATCGGTGCGGTTCGTTTCGGGTTA  GGTGACGCTTCAAAAGAAGATACATCTTTTAACGACATCAAACTTTCTAAATTATGGTTA  GGCTTAAAACATAACGATATCGGTAAAGTAACTTTCGGTAAACAAAATACCACTGCGGAT  GATGTTCAATTAAATGATCATACCTATATCTTTGGCGGTAACAACAACTTGGTTACTTCC  GGTGATAAAGTGGTGTCTTTCCGTACAGCAGATATCCAATTAGCGGAAGGGCAAACTTTA  GGTTTTGGTCTTGATTATGGTTTCGGTGAAGCACATAAAAAAGATATTAAGGATGGTGGA  CTTAAAGTAAGTGAAAAAAAACATATTAGGCTAAAAAATACTTACGGTCTCTCTGCATTC  TATACCGGCAATTTCGGTGATTTTACTGTAAATGCTAATGCCGGTTATACGGTTCATAAC  GAAAATACCATTCCTGGTCCAATTTACTCCAAAACCGATAATCAACAACAAGCTTGGCGT  TTAGCAACGCAAGTAGAATTCGGACCGGCATCTTTGGGTATTGAATACGGTCAAACAGTA  TATCAATCTAAAAAACAACATGAGTTTAATGGTTCCGCTCGCTTTGTTGAAGTTGGTGCA  AAATATGCAGTGTTACCGGATGTCTTAAATGTTTATGCACAATGGCAACGTAATAGTGTA  AGACAAGCTGTTGATAATATAGAGGGTAAATTTTCTCTTCCTTTCTCGCTCGGTGATGTA  AAAGTAGGTGATTTAAAACTTGGTGAACATAAGAAAGGAGTACAAAACGTATTTATCGTC  GGGGCTGACTATGCCTTTAACAAAAATCTATTAGCCTATGCAGAATTTGCTAATAGCCGT  GTTAAAGCACCTACAAAAGATAAAAACATAAGAGAAAGTTTTTACGCAGCAGGCTTACGT  GTTTACTTCTAA | MKKTLVALAVAAVAASANATTVYNQNGTKVEVGGRVDVMLGKFGDAQRTDLRNNGSRVEFKAEHEVQNGLKAIGAVRFGLGDASKEDTSFNDIKLSKLWLGLKHNDIGKVTFGKQNTTADDVQLNDHTYIFGGNNNLVTSGDKVVSFRTADIQLAEGQTLGFGLDYGFGEAHKKDIKDGGLKVSEKKHIRLKNTYGLSAFYTGNFGDFTVNANAGYTVHNENTIPGPIYSKTDNQQQAWRLATQVEFGPASLGIEYGQTVYQSKKQHEFNGSARFVEVGAKYAVLPDVLNVYAQWQRNSVRQAVDNIEGKFSLPFSLGDVKVGDLKLGEHKKGVQNVFIVGADYAFNKNLLAYAEFANSRVKAPTKDKNIRESFYAAGLRVYF |
| R15 (group C) | ATGAAAAAAAATTATTACACTGTACTTTCTCTGTCAATTTTGACCGCACTTTATAGCACG  TCCAGTCAAGCGAACTTACAACAGCAATGTTTGATCGGTGTTCCTCATTTTCAAGGTGAA  ATCGTTCAAGGCGATCCCAATGAATTGCCGGTTTATATTGAAGCAGATCACGCCAAAATG  AATCAATCGACACATGCTCAATACGAAGGAAATGTTAATGTTAAACAGGGCAACCGTCAT  TTAACAGCGGGAATGATTGAAATTGAGCAACACGGAAAAGATAATGCGAAACGTTATGCG  TATGCTAAAAATGGGTTTGACTACAAAGATAATTTAATTCAGCTCAATGGTGATAATGCT  AAAATTCACCTTGATAGCAAAGATGCCAATATCCAAGATGCAGATTATCAATTGGTTGGA  CGACAAGGGCGGGGAACTGCTGATGAAGTTGAACTTCGTGAACATTATCGAGTGATGAAA  AATGCAACTTTCACATCTTGTTTGCCTAATAGCGAAGCTTGGTCAATTGAGGCTAAGGAA  ATGCGTCAACATATTCAAGAAGAATATGCGGAAATGTGGCATGCTCGTTTTAAAGTATCC  GGTATCCCTATTTTCTACACGCCTTACCTACAATTACCTATCGGTGATCGCCGTCGATCT  GGATTACTTATTCCCAAAGCAGGTATCTCAACTCGGCATGGTTATTGGTATGCACAACCG  TTTTATTGGAATATAGCACCAAACTTTGATGCGACATTTACCCCTAAATATATGTCTCAT  CGAGGTTGGCAATTAAATGCAGAAACTCGCTATCTGACTCGTATCGGTGAGGGAAAATTT  GTCGTTGAATACTTAAAAACAGATCGTCATTCTGACTATTTAAATACGGCTCGTTCACGT  CATCTCTTTTATTGGGGACATAATTCTCATTTTCTAAAAGATTGGCGTTTAAATGTAAAT  TATACAAAAGTAAGCGATAAACATTATTTCAATGATTTTGAGTCTGAATATGGAAACAGT  ACAGACGGATATGTAGATCAACAGGCGAGCATTTCTTACTATCAACCGAATTACAACCTT  TCTATTTCAGCGAAACAATTCCAAATTTTCGATAAAGTAGATATTGGACCTTATCGTGCG  TTGCCACAAATTGATTTTAATTATTATCGCAACGAAATTGCTAATGGCTTAGTTGACTTT  AGTTTATTTTCACAAGTAGTTCGTTTTGATAATGACAGTGCGTTAATGCCAACTGCTTGG  CGTTTCCATATAGAACCGAGTTTGACTTTTCCACTTTCCAATCGTTACGGCAGTTTAAAT  ATTGAAACTAAACTTTACGCAACACGCTATCTACAAAAACGAGGTAAAGGAGAAAATGCA  GAAGAGATTAAAAAAACGGTTAATCGTGTTTTACCACAAATCAAGCTGGATTTTCAAACG  GTCTTAGCAAATAGACAAAGTTTCATTGAGGGTTATACCCAAACTCTTGAGCCAAGATTT  CAATATTTGTACCGCCCTTATAAAGATCAGTCGGATATTGGTCTAAAACAACAAAATAAT  GATTACTTAGGTTTTGGTTACGACTCAACTTTATTACAACAGGATTATTTTTCTTTGTTT  CGAGATCGCCGTTATAGCGGTTTAGATCGCATAGTTTCAGCAAATCAAATTACTCTTGGT  GGAACGACCAGATTTTATGATAAAAATGCAAATGAACGCTTTAACTTATCTATTGGACAA  ATTTATTACCTTAAAGACTCTCGCACAGATAATAATCCACAAAATATGGCTCAAGGCAGA  TCTTCTTCCTGGTCTTTAGAAAGTAACTGGCGTATCAATAGCAAATGGAATTGGCGTGGA  AGTTATCAATATGACACACATTTAAACCAAACATCTTTGGCAAATACCGTCTTAGAGTAC  AATTCGGAGAAAAATAACTTAATCCAACTCAGTTATCGATATGTTAACCAGTCTTATATC  GATCAAAATTTAATTGGTAAAAATACTTATGGACAAAGTATAAAACAACTTGGTATGACA  ACAGCTTGGGAGCTAACTGATCATTGGACACTGGTTGGTCGCTATTATCAAGATCTCGCA  TTGAAAAAGCCGGTTGAACAATATTTGGGAATACAATATAACTCTTGTTGCTGGTCTATA  GGTGTTGGAGCAAGACGTTATGTAACCAATAGAGCAAATCAACGCAATGATGAAGTGCTT  TATGATAATAGCTTAAGTCTCACTTTTGAGTTACGTGGATTATCTCCTTCAGATCATAAA  AATAATATAGATGAAATGCTGAAAAAAGGAAAACTGCCTTATATTAAAGCCTTTAGTCTA  TACTAACGTTCAAATTTAACGAAATATACCAAAAAACAAACCGCACGT | MKKNYYTVLSLSILTALYSTSSQANLQQQCLIGVPHFQGEIVQGDPNELPVYIEADHAKMNQSTHAQYEGNVNVKQGNRHLTAGMIEIEQHGKDNAKRYAYAKNGFDYKDNLIQLNGDNAKIHLDSKDANIQDADYQLVGRQGRGTADEVELREHYRVMKNATFTSCLPNSEAWSIEAKEMRQHIQEEYAEMWHARFKVSGIPIFYTPYLQLPIGDRRRSGLLIPKAGISTRHGYWYAQPFYWNIAPNFDATFTPKYMSHRGWQLNAETRYLTRIGEGKFVVEYLKTDRHSDYLNTARSRHLFYWGHNSHFLKDWRLNVNYTKVSDKHYFNDFESEYGNSTDGYVDQQASISYYQPNYNLSISAKQFQIFDKVDIGPYRALPQIDFNYYRNEIANGLVDFSLFSQVVRFDNDSALMPTAWRFHIEPSLTFPLSNRYGSLNIETKLYATRYLQKRGKGENAEEIKKTVNRVLPQIKLDFQTVLANRQSFIEGYTQTLEPRFQYLYRPYKDQSDIGLKQQNNDYLGFGYDSTLLQQDYFSLFRDRRYSGLDRIVSANQITLGGTTRFYDKNANERFNLSIGQIYYLKDSRTDNNPQNMAQGRSSSWSLESNWRINSKWNWRGSYQYDTHLNQTSLANTVLEYNSEKNNLIQLSYRYVNQSYIDQNLIGKNTYGQSIKQLGMTTAWELTDHWTLVGRYYQDLALKKPVEQYLGIQYNSCCWSIGVGARRYVTNRANQRNDEVLYDNSLSLTFELRGLSPSDHKNNIDEMLKKGKLPYIKAFSLY |
| R21 (group C) | ATGAAAAAAACAGCATTAGTACTTAGCGTGGCGGCTGCATTGGTGGCAGGAAGTGCAACA  GCACATGAAGCAGGCAGTTTTATTGTTCGGGGTGGTCCTATTTTAGTTGTGCCGAAAACA  TCAACAAACCATGATATGTTTAAGTTTGATGTAAACAAAAATGCCCAATTGGGGTTGACC  GGAACCTATATGATGACAGATAACTTCGGGGTTGAGTTATTAGCCGCAACACCGTTTCAT  CATAAAATCACGTTAGGCGACAAGCTAGTAGGTAAAACAAAACATTTACCGCCAAGCCTA  TATTTACAATATTATTTCTTAAACAAAGATTCTAAAGCACGTCCTTATATTGGTGCCGGT  GTGAACTATACTAAATTCTTTGGTGAAAAAGCGATAATGGAGGGTGTTTCCAATCTTAAA  TTAAAAGATTCTTGGGGAGCAGTATTTAACGCAGGGGTAGATATTCAATTGTCCGATAAT  CTTTATTTAAATACCGCTGTTTGGTATGCGAAAATTAAAACAAAAGCAAGTTTTAAAATG  AATGGTGCTGGTTCATCGGGGGGGGGTAGAGGAGTCCAACAACCTCAACAGAATAAAGTC  GATGTAACCCTTGACCCAATGGTATTTTTCATCGGTTTAGGTTGGAAATTCTAAGCAGAG  AAAAATATTGT | MKKTALVLSVAAALVAGSATAHEAGSFIVRGGPILVVPKTSTNHDMFKFDVNKNAQLGLTGTYMMTDNFGVELLAATPFHHKITLGDKLVGKTKHLPPSLYLQYYFLNKDSKARPYIGAGVNYTKFFGEKAIMEGVSNLKLKDSWGAVFNAGVDIQLSDNLYLNTAVWYAKIKTKASFKMNGAGSSGGGRGVQQPQQNKVDVTLDPMVFFIGLGWKF |
| R24 (group C) | ATGAAAAAAATGAAATTCACAAAAAACTTTTTTACGCCAACGCTTATTGCGTCAGCACTA  GCATTATCCACAACTTATGCCAATGCGGCAGCTTTCCAACTCGCCGAGGTGTCCAGTTCC  GGTTTAGGACGAGCTTATGCGGGTGAGGCAGCGATTGCTGATAATGCCGCTGTTGTGGCA  ACCAACCCAGCGTTAATGAGTTTGTTTAAAACGAAACAATTCTCAATGGGGGGGTATACG  TTCAATCTAGAATTAATATAAATGGTGAGGTTAATGTTAATGTAGGGAAGGGAATTGTAC  AAGTTGCAGATGAA | MKKMKFTKNFFTPTLIASALALSTTYANAAAFQLAEVSSSGLGRAYAGEAAIADNAAVVATNPALMSLFKTKQFSMGGYTFNLELI |
| R34 (group C) | ATGTCAGTTTATGATTTAAAAACTAAAAAAACCTTATTAGCTGTCAGCGTCTGTTTAGCG  TTTTCCGCTCAAGCAGAAACAAGTAAAAATAAAGTTGAACGAGCTAATCAATTACCGGAG  GTTGTTGTTTATGCAGAGCAAAACGCAGGATTATCTTCCAGCCAAAAAGTAACCGCCAAA  GATATTAAATCTTCCCCTAATTCCAACGGCAATATTTCTGATTTTTTGAAAACCAATTCT  CATGTGCGTTTTGAACGTAGTGATGAAAACAGTTTTCAACGAGGTGAAATTAAACCTGCT  GACATTTCGATTAATGGTGCGGAAGCCAGTCAAACCAGTTATTTTGTAGATAATGTCAAT  ATCAATAATGATTTAGGATTTGACTCTGAAATTTTTGAGGGAGCGATGCAAACTTTGCCT  ATGGCAAGTCATGCACAAGCCTATTTCTTTGATGCGAATTTATTGTCTTCCGTAACCGTT  TACGACAGCGATATTTCCGCCAGTTTAGGCGGTTTTGCCGGCGGTGCCGTAGTCGCCAAA  ACCAAGCAATATGACGGTACAGATGGTGTGCAACTGCGTTATCGTACCAGCCATTCTAAT  TGGGCGAAATTCCATCTTGAGGAAAAAGATCGAGAAAAATTTAAACAAGCCTCGCCTAAC  GGTAGTAGTGCGGATTTTCAACCTAAATATAGCAAAGATTTCTTCAGCCTTTCTGCACAA  CATTCTTTAGGGGAAAATATCGGTATGGTAGCAGGATTTAGTCGCCGTACTTCAGATATT  CAGCAACGTCGTTTAGTGCTGGGAAAAGATAACAAGTTGAGTTCGGATAATCGCAGACAT  AAACGCCGTTCCGATAATGCGTTGTTGAATTTTAACTGGCTTGCTAATGAGGATAATCGC  TTTGAATTGAGTTTGCGTTATTCTAATTATGTGGAAACTAAATTCTTTGCAGAGAATGTT  GATAGCAATGTGCAAGATTATCATCAAGCCTATGGTGCTACTTTAGCTTGGATTCGTTCG  TTAAAGAGCGGTGTGTTAACTAATACATTGGCGTATGATCAATTTGCAGATAAGCGTAAA  TCCGCCTCTAATTATTTGAAGCAAATATTGGCATTTGATGAAAACTATGATCCGATTAAT  TATGAACGTGGAGGAATGGGAGATAGTGCTTTAACGCAACGCAATGTGCATTTTTCCAGT  GAATTTGCTATGGATCCGTTGACTTGGGGACGTACTGAACATTCTATTTCCTTAGGGGGT  ATCTGGCAATTTACGCACTATCGTTTTCAACGTGATCAAAATGCTAAATCTGAAATTTTC  ATGCAGGACAGTATGGAAAGTCCTCTTTCTTCAAATTCCGTTTCCAAAGGAACAGTAAAA  ACCGATTATCACAATATCGCCCTTTATGTCGAAGATTTAATCAAGCTGGGGGGGGGGAAA  TTGGGAGTTTCGTCCGGGGTTACGTTTAGAGCGTGATAATTTCCTTAAAAATACCAATAT  TGCCCCTCGTT | MSVYDLKTKKTLLAVSVCLAFSAQAETSKNKVERANQLPEVVVYAEQNAGLSSSQKVTAKDIKSSPNSNGNISDFLKTNSHVRFERSDENSFQRGEIKPADISINGAEASQTSYFVDNVNINNDLGFDSEIFEGAMQTLPMASHAQAYFFDANLLSSVTVYDSDISASLGGFAGGAVVAKTKQYDGTDGVQLRYRTSHSNWAKFHLEEKDREKFKQASPNGSSADFQPKYSKDFFSLSAQHSLGENIGMVAGFSRRTSDIQQRRLVLGKDNKLSSDNRRHKRRSDNALLNFNWLANEDNRFELSLRYSNYVETKFFAENVDSNVQDYHQAYGATLAWIRSLKSGVLTNTLAYDQFADKRKSASNYLKQILAFDENYDPINYERGGMGDSALTQRNVHFSSEFAMDPLTWGRTEHSISLGGIWQFTHYRFQRDQNAKSEIFMQDSMESPLSSNSVSKGTVKTDYHNIALYVEDLIKLGGGKLGVSSGVTFRA |
| R36 (group C) | ATGAAACTCACTTTATCAGCCATTCTTCTTCTTTTTCCGGTTTCCGTACTGGCGCACAGT  CCCAAAAGTCCGAGCGAACATTTGGACGATCACCGCATTGCAGATGAGCGGGTACGTGAA  AACATTCAAGATGCCTTGCCGACACAACCTAAACAAACCGTTGTGCCGAACATTCAGCCA  CAGCAAACGGTTGCATTAAGCGAAAGCCAGTTACAGCAACATCCTGATTTACTTGAGCGT  GCATTGATAGCGGCTTTATTGCAAGGTAATGGTGAGAATGCCTCTTTGCTGTTACCGCAC  TATCAAAAGTTGCCTGAAAACCTGCAAGAGCCCACTTTTCACCTTTGGGCAAAAGCCTTG  ATTGCGCGTTGGCGTCATCAATATACACAGTCCGTGCGTTTATACCGCCAAGCCTTAGCA  CAACAACCTGATTGGTCGGTTTTGCGTTTACAGACCGCCGCCGCCCTCTTGTCCAATAAA  GAATTCGATGCGGCAGAAGCTCAGTTTCGCAAGGTGCAAAGCGAAAATCAGCTACCCGCC  GGATTTGCCCAAGAAATTGAATCAGTTTTGCTGTATATCAAACGGCAAAGCCGTTGGCAA  TTCAGTGGCAACACTACCTATATTAACGACAAAAATATTAACAACGCTCCTAGAAATCCT  GATTTGGGCGGAGGTTGGCGGGGTGATCAGGCTGAGTCCGGTCAAGGGTTAGCCGTTAAT  TTGGGCACCAATAAAAAATGGTTTTGGAAAAATGGATTATTTAATGAATGGCGTTTAGAC  AGCAACAGTAAATTTTATTGGAACAACAAACGATTCAACGAAGCCAATGTGCGTGCTTCA  ATGGGTATCGGTTATCAAAATGCTAAAAATAGCATTACCGTTCTGCCATTTTTTGAGCAA  GCGTGGTATGCAGGGGGCAAGAAAGGCAATGAGACCTTACGACGTTTTTCCAACAGTCGA  GGTATTGCACTAGAAGCAACCCACACTTTTAGCCCCAAATGGCAGGGGAGTCTGACAGCT  GAGACGGCACAAAATCGTTATCGGACACGTAAGCATTTAAACGGTAATACGCACTTTGTT  TCTTTATCGGCGGTGTATCAACACAATCCGAGTCAAGCTTGGTTTGGAGGAATAGACTGG  CATCGCAACAACGCACGAGATGGCGATGATTCTTTTGACCGTATCGGGGTACGGGCAGGC  TGGTTGCAAGACTGGAAAGGACTTTCCACACGCTTAATTACTTCTTATGGCAAAAAAAAC  TATCGCAGTGCAGGCTTTTTCAACAAAACCCAACGTAATCGAGAGTTGGGCGTACAGGTC  AGTGTATGGCATCGAGCCGTACACTGGCAAGGTTTAACACCACGGTTAACATGGTCATAC  ACTAAAACGGATAGTAACATACCATTGTTCCGTTACAACAAACAGCGCCTGTTTCTGGAA  ATTAATAAGCAGTTTTGA | MKLTLSAILLLFPVSVLAHSPKSPSEHLDDHRIADERVRENIQDALPTQPKQTVVPNIQP  QQTVALSESQLQQHPDLLERALIAALLQGNGENASLLLPHYQKLPENLQEPTFHLWAKALIARWRHQYTQSVRLYRQALAQQPDWSVLRLQTAAALLSNKEFDAAEAQFRKVQSENQLPAGFAQEIESVLLYIKRQSRWQFSGNTTYINDKNINNAPRNPDLGGGWRGDQAESGQGLAVNLGTNKKWFWKNGLFNEWRLDSNSKFYWNNKRFNEANVRASMGIGYQNAKNSITVLPFFEQAWYAGGKKGNETLRRFSNSRGIALEATHTFSPKWQGSLTAETAQNRYRTRKHLNGNTHFVSLSAVYQHNPSQAWFGGIDWHRNNARDGDDSFDRIGVRAGWLQDWKGLSTRLITSYGKKNYRSAGFFNKTQRNRELGVQVSVWHRAVHWQGLTPRLTWSYTKTDSNIPLFRYNKQRLFLEINKQF |
| R1 (group D) | ATGTTTCCTATTATTAAAATTTCATTGAAATACTCATCTTAACATTACGTCCAACACCTGAAACTTTTTCACTTAGATAAGGGTTGTACTTGCGATTAAAGATATTGTCTATGCTTAGAGAAAGCTTCATTCCGTGTATTTTCTTAGGTTCCCATATGGCAAATAGATGATGTAATGAGTAGCCGTTGGATTTAGGAAGTGACCAATACCCTGCTTCAGGATCGTTATCGATAGGTGATCTATCTTGGCGACGGAAAAACTCACCTTGCCAACCAATTGATATATTCCATTTGGGTAGGTTTGTACCTAATGTAATTATTGCCTTACGAGGAGGGATCTCTGCTATCCAAGTTTTGCTGGCAAAATTAGGATCTCTAGGAGATGCCTCTCGTTTACCTTTAATGTACGAATAAGCGATGCTACCAAACAAATATTTAGACTCATAATAAGCTTCTAAATCTAAACCATAAATGTCATAACCGGGTAAATTACGATAATTACTGATAGGTTTGGTATCATGAACCACACCTCTTGTCTTGAATATTTCGTCTTGACCATGATTATTAAATGCTGTGGTTCTAAACTGTAAATAGTCATTGTCAATCAATAGATTTGTAAAATTCAGGATGGCTCCAAACCTTAAGGCATTTAAATTTTCTTTATCCAAATTACGGCTTGTACCGGTAATTTTTGCTTTGGCATATTGTACTTCATATTGTTCATCAATTACAGGAGCACGCCATGAGCGACCGAAATCACCGAAGAAACGAAAATTATCTGAAATTTTCCATTGCAAACCTATATACGGCGACCATCCGGTATAGGTTTTTGCTGTATAATCGTGCCCTGCCAGTAAATTATTATATATTGGAGCAATATTTTCTTTACCCACATTACGAACATGGTCATAACGCAGTCCTAATTTGATGATGAAATTATTTAATTCCATGTTATCTTCAATAAATATACTGCGTCTATATTGTACTCCAGATGGCATATAATAAGGCTGATACCAACCAAAATTATACTCTGCACGTGTCGATTTTGAGGGATCCCACATCATGATATCTCGTTTACCCCGATGATATTGTATTCCTGTCTGTAAGGTATGTAGCACAATACCGGTGGCAAACTGTGACGTATTATTTAATTCAAATTGAGTATCTTGATATACTGTGTTGCTTGCATTTCCCATACTGGCAACAAAGAACTTGGCAGCCTCTTTATGACGTTTATCCACTTGTTTTGTTCTTGCCCATCCAATTTTTGCTTCTAGATTAATTAAAGGATGATCTGATATGTAAGTCCAGTCTAGAGCAACACTTTCATCAGTCAGTTTGCGATATACCAGTTTCCTTTTCCAAGCAATATCTTCTCCCCATTTCTTTATGTCGGATATACTTGGTGAGGGTAAATTATCACGCATAGCGGCAAATGGTGTCCAACCAGAGTTATTGGAGCGTACAGCTGAAAGCGTCAATTGTTGCTTATCTGAAGGATAAAAATTGACTTTTAATAGATAGCTATTATTGTCATTTTCAGAGTATAAGAAACGAGAACCATCTGGGCGTTTTATGTTATGACTATTACGAACGGTTGTGTAAAATAAGCCCTCTAGATTTTTTTCTTGATTGCGTAGAAAAATTGCACCGCTCCAAATATTCTGTTTATCATTCGTATGGTAACTATATTTAGCAAATCCACCAACATTTTGATTTTCATTGAGAAAGTCGCTCGGATTTTTAGTCATAAATTTTAAGGTTCCGCCAAATCCGCCATTCCCATATTTTGCAGAAAAGCTGCCTTTATCTACTTCCACCCGACGCAGTAATTCCGGTTCTATAAATACAGATCCTTGACGATATCTTTCAAAGTTTTTTTGTATACCGTCTAATTCAATACGGACATCTTCACTATCACCAAATCCCCAAATATTGATATTTTGACCTCCCGGTCTAGGTGAACCTGACATATTGACACCAGGCAAAACATTAACTAAGGCTGCGGCATTGTCAGCTTGCTGTCGGTCAATATTTTTTGTTAATAGTGTTGAACGTCCGATATCATACTGTTCATTTTGAACTAAGATGACAGGAAGAGCGTCGTATTGTTGAGCGATTAATTTGCTTGTATAAAACGTAGATGAAAGCAA | MLSSTFYTSKLIAQQYDALPVILVQNEQYDIGRST  LLTKNIDRQQADNAAALVNVLPGVNMSGSPRPGGQNINIWGFGDSEDVRIELDGIQKNFERYRQGSVFIEPELLRRVEVDKGSFSAKYGNGGFGGTLKFMTKNPSDFLNENQNVGGFAKYSYHTNDKQNIWSGAIFLRNQEKNLEGLFYTTVRNSHNIKRPDGSRFLYSENDNNSYLLKVNFYPSDKQQLTLSAVRSNNSGWTPFAAMRDNLPSPSISDIKKWGEDIAWKRKLVYRKLTDESVALDWTYISDHPLINLEAKIGWARTKQVDKRHKEAAKFFVASMGNASNTVYQDTQFELNNTSQFATGIVLHTLQTGIQYHRGKRDIMMWDPSKSTRAEYNFGWYQPYYMPSGVQYRRSIFIEDNMELNNFIIKLGLRYDHVRNVGKENIAPIYNNLLAGHDYTAKTYTGWSPYIGLQWKISDNFRFFGDFGRSWRAPVIDEQYEVQYAKAKITGTSRNLDKENLNALRFGAILNFTNLLIDNDYLQFRTTAFNNHGQDEIFKTRGVVHDTKPISNYRNLPGYDIYGLDLEAYYESKYLFGSIAYSYIKGKREASPRDPNFASKTWIAEIPPRKAIITLGTNLPKWNISIGWQGEFFRRQDRSPIDNDPEAGYWSLPKSNGYSLHHLFAIWEPKKIHGMKLSLSIDNIFNRKYNPYLSEKVSGVGRNVKMSISMKF |
| R4 (group D) | ATGGCTACTGTTAACAGAGGATCGCATCACCATCACCATCACAACAGATCCATGTTAAAA  CTTTCTAAAATGACGTTTGCAATAGTGGTGTCTAATATATTAATCGGTTGTGCAAATATT  GATGATAGTTACCATGCAACATTACAAGATTTTCAGCAATATGAAGCATTGACTCAACAA  TATAACATTCAGGAAAATTGGTGGACACAATATAATGACGAACCGCTTAACCAACTGGTG  AAACAAGCTTTGGAAAATAATAAAGATCTTGCCAAAGCCACTATTGCAGTAAACACGGCT  CTGTACAATGCCAATTTGGTTGCGGCGGATTTAGTACCAAATTTCACACGCCAAACAGGC  GTTAATTCTTCAGCTGCCAAAAATATTAACACAGGGAGCAACTCTAAAATTACTCACGGC  GGTTCACTCAATATTAGCTACACCCTAGATTTATGGCAACGTTTAGCCGATAAAACCTCT  GCTGCAAAATGGACACATTCCGCTACGCAACAAGATTTACAAGCAACAAAACTCTCTCTG  ATCAACTCAGTTGTCACAACTTACTATCAACTTGCCTATTTAAATGATGCCATTGCAGTA  ACAAACCAAACGATTAATTATTACTCAAAAATTAGTCGAATTATGCAAAATAAATTCAAG  CAAGGTGTAGCAGATCGGGCAACTACAGATCAATCACAACAATCAATTCTTAAGGCTCGT  AATAATCTGATTAATTATCAAACACAAAAAAAACGTGCCGAAACTATCCTGCGTAATTTG  CTTAACTTAAAACCGAATGAAGATTTAAACATCAAAACACCAAATATCTTGGAAGTCAAA  AGTGCGGAGGTAAATTTGGATATTCCATTATCAACCATTGCCAATCGTCCGGATATTAAA  GGCTCTTTATATCGCCTCAATAGTGCATTTAAAAATGCGAAAGCAATGCAAAAAAGTTGG  TTTCCATCAATCACACTGGGAGCAGAATTATCATCAAGCGGTAATAAAATTGACAATGCT  TTCAACATACCGATTGCGACAGGAACGGTAGGCATAAACTTACCTTTCTTAAATTGGAAT  ACAGTAAAATGGAATGTTAAAATTTCTGAGGCAGCTTATGAAAATGCAAAACTGAGTTTT  GAACAAAACATCACCAAGGCTCTAAATGAAATAGACAACCTCTATTTCTCTTATACCCAA  GCAAAAGAAAATGTTGCAAATTTACAAGAGACTTACCGTTACAACAAACGTATTACGCAA  TATTACAAAAACCGCTATGATGCCGGTATCGCAGAATTAATAGAATGGCTTAATGCCGCT  AATACAGAAAATACCTCTCAATTAGCCATTCTGAATGCGAAATACAGTTTAATTCAAAGT  CAAAATGCGATTTACAGTGCGATGGGAGGATATTATTCTACTAATTCCCCTCGCTAA | M A T V N R G S H H H H H H N R S M L K L S K M T F A I V V S N I L I G C A N I D D S Y H A T L Q D F Q Q Y E A L T Q Q Y N I Q E N W W T Q Y N D E P L N Q L V K Q A L E N N K D L A K A T I A V N T A L Y N A N L V A A D L V P N F T R Q T G V N S S A A K N I N T G S N S K I T H G G S L N I S Y T L D L W Q R L A D K T S A A K W T H S A T Q Q D L Q A T K L S L I N S V V T T Y Y Q L A Y L N D A I A V T N Q T I N Y Y S K I S R I M Q N K F K Q G V A D R A T T D Q S Q Q S I L K A R N N L I N Y Q T Q K K R A E T I L R N L L N L K P N E D L N I K T P N I L E V K S A E V N L D I P L S T I A N R P D I K G S L Y R L N S A F K N A K A M Q K S W F P S I T L G A E L S S S G N K I D N A F N I P I A T G T V G I N L P F L N W N T V K W N V K I S E A A Y E N A K L S F E Q N I T K A L N E I D N L Y F S Y T Q A K E N V A N L Q E T Y R Y N K R I T Q Y Y K N R Y D A G I A E L I E W L N A A N T E N T S Q L A I L N A K Y S L I Q S Q N A I Y S A M G G Y Y S T N S P R |
| R22 (group D) | ATGAAGAAAAAAATCACTTTTACTAAATCTCTCATCGCTTCAGCGTTGGCATTATCAGCG  AGTTATGTCAATGCGTCTGCTTTCCAACTCGCCGAGGTATCCAGTTCCGGTTTGGGGCGT  GCTTATGCAGGTGAAGCGGCAATTGCTGATAATGCCGCCGTTGTAGCAACCAACCCTGCA  TTAATGAGTTTGTTTAAAGCGAAACAATTCTCATGGGGGGGGGGTATACGTTGA | MKKKITFTKSLIASALALSASYVNASAFQLAEVSSSGLGRAYAGEAAIADNAAVVATNPALMSLFKAKQFSWGGGIR |
| R23 (group D) | ATGAAAAATGCAGTAAAACTGACCGCTCTTTCTATCGCATTAGGCTCATCAATTGCAATG  GCAAGTGATAATATCGCTGTTGTTAATACAGAGTACTTGTTTTTAAATCACCCTGCTCGC  TTGCTTGAGTTTCAAAAATTAAATGAGGAATTTAAAGCACCAGCAGAAAAACTGGAAGCA  GCAGATAAAGCATTAGTTGAAAAACGTGACAATTTTGAAAAAGAAATTGAAACAAAAAGT  AAAGCATTGGAAAAAGATGCACCGAAACTACGTCAAGCTGATATCAAAAAACGTCAAGAT  GAAATTGCTAAATTGGTAACAAAACGTAATGACGAGTTTAACAAGCTGGTAGCGGAGCAC  CAAAAAAATGTTGCAGCGTTTCAAGCTGAAGCACAAAAACGTGATGCCGAAATTACTCAA  CGTTTATTAAAAGAGGTTCAAACAGCTACAACGAATGTAGCTAAAGCCAAAAACTTTACA  ATTGTATTGGATGAAAAAACGGCAATTTATGTTGCTGACGGTAAAAATATTACCGAGGAA  GTTCTAAAAGCTATTCCGGCTCCAGCTCCAGCAAAGGCTAAGTAATGCAAGGATATTCTT  TACTGGAATTGGCTCAACAAATCGGCGCTACCATTCG | MKNAVKLTALSIALGSSIAMASDNIAVVNTEYLFLNHPARLLEFQKLNEEFKAPAEKLEAADKALVEKRDNFEKEIETKSKALEKDAPKLRQADIKKRQDEIAKLVTKRNDEFNKLVAEHQKNVAAFQAEAQKRDAEITQRLLKEVQTATTNVAKAKNFTIVLDEKTAIYVADGKNITEEVLKAIPAPAPAKAK |
| R35 (group D) | ATGAGATACAAATTATCTTTAGTACTTTTATCAATGCTGCCCGCACTTTCCCATGCGGGG  GGGGGGGGGAATTTCAACTTTTACGGCAAAGCGGGAATTGATTTAACCTCTCGTTTTGAA  ACGATGAAAATAACACAAAATTACGATAGTGCTGGCGGTTTTAAATTATCAATTCCGGCA  AGTTCAAGACAAAACACTTTCTCACCCAGTATCTTCTTTGAAACGACTTACAATATTTTT  CCGCAAACGGAAATTGGTCTCGGACTAGGCTATATCAAACGAAAAGGCTTTGACCATATT  GCAATTTGGCCCGTTAGAGGGGGTGCTGAATACGATCCGAGCGGACAGTTTCATCTGAAA  GAAACCTATAGAGTAAATCGTTATGCTTCCCTACCAATTTATTTTATTTTGAAACAAAAT  TATGCCTTAAATGCAAATACAAAATTTTATCTAAAAGGCGATTTAGGGTATTCAATTAAT  AAAATTCGTAAAACGGTGTATACCTCATATGGAGATATTGGAACAACTGCAGGAGTATAT  GAGGAATCTTATAAATTTGTTAGTAATAATAAAGCAAAGAGCGGTCTGTATTTAGGCTTA  GGTATTGGGATTGAATATAAATCTTTCTTAGTAGATATTGGATATTATCATACCCATTCG  AAAATCACTTATAAAGAACAAGAAAGTTACGTATCAACACCCTATAACAACGATGCAATA  CGCTTAACGCTTGGCTTTAAATTCTAA | MRYKLSLVLLSMLPALSHAGGGGNFNFYGKAGIDLTSRFETMKITQNYDSAGGFKLSIPASSRQNTFSPSIFFETTYNIFPQTEIGLGLGYIKRKGFDHIAIWPVRGGAEYDPSGQFHLKETYRVNRYASLPIYFILKQNYALNANTKFYLKGDLGYSINKIRKTVYTSYGDIGTTAGVYEESYKFVSNNKAKSGLYLGLGIGIEYKSFLVDIGYYHTHSKITYKEQESYVSTPYNNDAIRLTLGFKF |
