# Supplementary material for: Cattle Immunized with a Recombinant Subunit Vaccine Formulation Exhibits a Trend towards Protection against Histophilus somni Bacterial Challenge
Source: PLoS One. 2016 Aug 8;11(8):e0159070. doi: 10.1371/journal.pone.0159070 (PMC4976985; doi:10.1371/journal.pone.0159070)
Supplement: S2 Table — The vaccine for group A (placebo/control group) did not contain antigens. The vaccine for groups B, C and D contained antigens (R2, R5, R8, R18, R27, R37), (R13, R15, R21, R24, R34, R36) and (R1, R4, R22, R23, R35) respectively. (DOCX) [file pone.0159070.s002.docx]

**S2 Table**. Statistical determination of serum antibodies using Mann Whitney test

| **Statistical analysis of ELISA results** |  |  |  |  |  |  |  |  |  |  |  |  |  |  |  |  |  |  |  |
| --- | --- | --- | --- | --- | --- | --- | --- | --- | --- | --- | --- | --- | --- | --- | --- | --- | --- | --- | --- |
| Vaccine group | **B** | **B** | **B** | **B** | **B** | **B** |  | **C** | **C** | **C** | **C** | **C** | **C** |  | **D** | **D** | **D** | **D** | **D** |
| Antigens | **R2** | **R5** | **R8** | **R18** | **R27** | **R37** |  | **R13** | **R15** | **R21** | **R24** | **R34** | **R36** |  | **R1** | **R4** | **R22** | **R23** | **R35** |
| **Mann Whitney test** |  |  |  |  |  |  |  |  |  |  |  |  |  |  |  |  |  |  |  |
| P value | 0.0019 | 0.0002 | 0.0006 | 0.193 | 0.0006 | 0.0003 |  | 0.0003 | 0.0011 | 0.0002 | 0.0003 | 0.0148 | 0.0002 |  | 0.0379 | 0.0002 | 0.0003 | 0.0006 | 0.0499 |
| Exact or approximate P value? | Exact | Exact | Exact | Exact | Exact | Exact |  | Exact | Exact | Exact | Exact | Exact | Exact |  | Exact | Exact | Exact | Exact | Exact |
| P value summary | ** | *** | *** | ns | *** | *** |  | *** | ** | *** | *** | * | *** |  | * | *** | *** | *** | * |
| Significantly different? (P < 0.05) | Yes | Yes | Yes | No | Yes | Yes |  | Yes | Yes | Yes | Yes | Yes | Yes |  | Yes | Yes | Yes | Yes | Yes |
| One- or two-tailed P value? | Two-tailed | Two-tailed | Two-tailed | Two-tailed | Two-tailed | Two-tailed |  | Two-tailed | Two-tailed | Two-tailed | Two-tailed | Two-tailed | Two-tailed |  | Two-tailed | Two-tailed | Two-tailed | Two-tailed | Two-tailed |

“R” is denoted for rank of gene or protein. The vaccine for group A (placebo/control group) did not contain antigens. The vaccine for groups B, C and D contained antigens (R2, R5, R8, R18, R27, R37), (R13, R15, R21, R24, R34, R36) and (R1, R4, R22, R23, R35) respectively.
