# Supplementary material for: Cattle Immunized with a Recombinant Subunit Vaccine Formulation Exhibits a Trend towards Protection against Histophilus somni Bacterial Challenge
Source: PLoS One. 2016 Aug 8;11(8):e0159070. doi: 10.1371/journal.pone.0159070 (PMC4976985; doi:10.1371/journal.pone.0159070)

**S3 Table.** The sum of joint lesions for each animal measured from day 1 to 21 post challenge for all groups: A (non-vaccinated) and B, C, D (vaccinated).


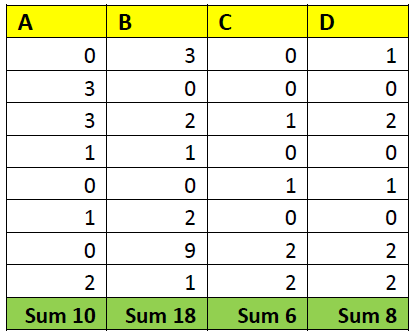

Supplement: S3 Table — (DOCX) [file pone.0159070.s003.docx]
