# Supplementary material for: Cattle Immunized with a Recombinant Subunit Vaccine Formulation Exhibits a Trend towards Protection against Histophilus somni Bacterial Challenge
Source: PLoS One. 2016 Aug 8;11(8):e0159070. doi: 10.1371/journal.pone.0159070 (PMC4976985; doi:10.1371/journal.pone.0159070)
Supplement: S4 Table — Bacteriological analysis carried out on samples of heart, lung, kidney and joints. Positive cultures for H. somni are shown in dark green. (DOCX) [file pone.0159070.s004.docx]

**S4 Table.** Full post-mortem analysis of euthanized animals. Bacteriological analysis carried out on samples of heart, lung, kidney and joints. Positive cultures for *H. somni* are shown in dark green.

|  |  |  |  |  | **Culture** | **Culture** | **Culture** |  |
| --- | --- | --- | --- | --- | --- | --- | --- | --- |
| **Calf ID number** | **Group** | **Number of Joints with lesions** | **Number of lesions in Kidney** | **Number of lesions in heart** | **Joint** | **Heart** | **Kidney** | **Clinicals** |
| 8 | A | 3 | 1 | 0 | Neg | Neg | Neg |  |
| 10 | A | 3 | 0 | 0 | Neg | Neg | Neg | Pleuritis |
| 13 | A | 1 | 0 | 0 | Neg | Neg | Neg |  |
| 27 | A | 1 | 0 | 0 | Neg | Neg | Neg |  |
| 34 | A | 2 | 0 | 0 | Pos | Neg | Neg |  |
| 6 | B | 3 | 0 | 0 | Neg | Neg | Neg |  |
| 16 | B | 2 | 0 | 0 | Neg | Neg | Neg | Pleuritis, Broncho pneumonia |
| 19 | B | 1 | 0 | 1 | Neg | Neg | Neg |  |
| 22 | B | 0 | 0 | 1 | Neg | Pos | Neg |  |
| 28 | B | 2 | 0 | 0 | Neg | Neg | Neg |  |
| 30 | B | 9 | 1 | 1 | Neg | Neg | Pos |  |
| 39 | B | 1 | 0 | 0 | Neg | Neg | Neg |  |
| 15 | C | 1 | 0 | 0 | Neg | Neg | Neg |  |
| 20 | C | 0 | 0 | 1 | Neg | Neg | Neg |  |
| 25 | C | 1 | 0 | 0 | Pos | Neg | Neg | Pleuritis |
| 29 | C | 2 | 0 | 0 | Neg | Neg | Neg |  |
| 40 | C | 2 | 0 | 0 | Neg | Neg | Neg |  |
| 3 | D | 1 | 0 | 0 | Neg | Neg | Neg |  |
| 14 | D | 2 | 0 | 0 | Neg | Neg | Neg |  |
| 21 | D | 1 | 0 | 0 | Neg | Neg | Neg |  |
| 36 | D | 2 | 0 | 0 | Neg | Neg | Neg |  |
| 37 | D | 2 | 0 | 0 | Pos | Neg | Neg |  |
